# Evaluation of mechanical properties and corrosion resistance of various coating systems used on tool steel in the mould industry

**DOI:** 10.1098/rsos.251931

**Published:** 2026-09-09

**Authors:** Mateusz Czepiel, Edyta Kosińska, Julia Sadlik, Agnieszka Sobczak-Kupiec

**Affiliations:** ^1^ CUT Doctoral School, Cracow University of Technology, Kraków, Poland; ^2^ Faculty of Materials Engineering and Physics, Cracow University of Technology, Kraków, Poland

**Keywords:** PVD, coatings, tool steel, balinit

## Abstract

This paper presents a comprehensive assessment of the surface and functional properties of selected tool steels (1.2343, 1.2083, 1.2378 and Toolox 44) coated with BALINIT LUMENA, BALINIT CROVEGA, BALINIT CROMA PLUS and BALITHERM PRIMEFORM protective coatings. The research aimed to determine their suitability for industrial applications, in particular in polymer injection moulds exposed to high temperatures, pressure and corrosive environments. The evaluation included microhardness analysis (HV1), surface roughness measurements, morphological observations using scanning electron microscopy (SEM) and Keyence optical microscopy, as well as tribological tests. In addition, techniques such as ellipsometry and reflectance angle analysis were used to characterize the surface properties of the coatings in detail. To determine chemical stability, corrosion resistance tests were performed under hydrothermal incubation conditions (80°C, distilled water, 14 days) and the chemical composition of the substrate was analysed using X-ray fluorescence (XRF). Comprehensive testing has shown that the correct choice of protective coating is crucial for the durability and reliability of moulds in injection moulding processes. In particular, the BALINIT® CROMA PLUS coating, thanks to the synergy of high microhardness, corrosion resistance and stable optical and tribological properties, is the most promising solution for industrial injection moulding applications.

## 1. Introduction

In response to the increasing technological demands of the injection mould industry, the selection of appropriate tool steels and their surface modification have become a critical factor in ensuring durability and performance [1–3]. Injection moulds used for processing polymer materials are exposed to extreme operating conditions—high temperatures, pressures and chemically aggressive environments. Consequently, the longevity and reliability of mould components largely depend on the protective coatings applied to their surfaces [4,5].

The use of coatings based on plasma arc evaporation began in the nineteenth century. The invention of the first vacuum pump to generate plasma by Michael Faraday in 1838 paved the way for the development of this technology. In 1852, William Robert Grove was the first to atomize the tip of a wire placed in a vacuum and deposit it on a polished silver surface [6,7].

Subsequently, in 1858, Thomas Edison created a device called the ‘Electrical Deposition Apparatus’, which was used to produce mirrors and became the forerunner of modern vacuum coating systems [8–10]. Since these discoveries, physical vapour deposition (PVD) processes have found diverse industrial applications—from military (e.g. gun barrels), through industrial (e.g. cutting tools, surgical instruments), to decorative uses (e.g. consumer goods) [7,11,12].

PVD coating technology involves altering the molecular properties of a metal surface by depositing thin layers of material. The unique combination of appearance and functionality makes this process a cornerstone of modern surface engineering [13–18].

In recent years, thin-film coating technologies, such as PVD and chemical vapour deposition (CVD/PACVD), have gained widespread application in surface engineering [19–22]. Coatings based on TiAlN, CrN and diffusion systems like PRIMEFORM significantly enhance the mechanical, tribological and corrosion-resistant properties of tool steels without compromising their structural integrity [6,21,23–25].

This study presents a comprehensive evaluation of the functional properties of four commercial protective coatings: BALINIT LUMENA, BALINIT CROVEGA, BALINIT CROMA PLUS and BALITHERM PRIMEFORM, applied to four grades of tool steel: 1.2343, 1.2083, 1.2378 and Toolox 44. The investigation includes microhardness testing (HV1), surface roughness measurements (Ra), morphological analysis (SEM and optical microscopy), tribological performance (coefficient of friction (COF)), corrosion resistance (hydrothermal incubation) and optical characterization using variable angle spectroscopic ellipsometry (VASE) [26–28].

Numerous studies have shown that the corrosion resistance of metallic coatings is strongly dependent on deposition process parameters, which influence the chemical composition, microstructure and uniformity of the protective layer. These properties are often analysed using electrochemical methods, such as potentiodynamic polarization and electrical impedance spectroscopy, supplemented by microstructural studies using SEM, X-ray diffraction and chemical composition analysis of the coatings [29–31].

The results enable the identification of the most effective coating system for industrial mould applications, considering both mechanical and aesthetic requirements, such as gloss. Particular attention is given to the synergy between coating properties and substrate microstructure, which is a key determinant of coating performance under industrial conditions.

Despite numerous studies on PVD coatings applied to tool steels, most of them focus on the analysis of a single coating system or a single type of substrate material. Under industrial conditions, however, the effectiveness of coatings depends to a large extent on the interaction between the chemical composition of the coating and the microstructure of the substrate material.

The significance of this work lies in providing a systematic, multi-parameter evaluation directly relevant to industrial mould design. The selection of injection moulds as the target application is motivated by the increasing use of engineering polymers processed at elevated temperatures and pressures, where coating degradation directly translates to reduced mould lifespan, surface quality loss of moulded parts and increased maintenance costs. The four steel grades selected (1.2343, 1.2083, 1.2378 and Toolox 44) represent the principal substrate materials used across different mould categories in European industry—from hot-runner systems operating at 200–350°C (1.2343) to high-gloss optical moulds requiring corrosion-resistant stainless substrates (1.2083). The four coatings (BALINIT LUMENA, CROVEGA, CROMA PLUS and BALITHERM PRIMEFORM) were chosen to cover the full range of commercially available Oerlikon Balzers systems recommended for plastic processing, enabling a complete comparative framework under identical test conditions.

In addition, the study employed a set of complementary material characterization methods, including SEM–energy-dispersive X-ray spectroscopy (EDS) analysis, surface roughness measurements, Vickers microhardness testing, tribological tests, corrosion tests under hydrothermal incubation conditions, X-ray fluorescence (XRF) chemical composition analysis and spectroscopic ellipsometry. Such a comprehensive approach allows for a better understanding of the synergistic influence of the coating structure and the substrate material composition on the functional properties of injection mould components used in industry.

The results obtained also have significant practical implications for the tooling industry. Injection moulds used in plastics processing operate under high temperatures, heavy mechanical loads and in environments conducive to wear and corrosion. Under such conditions, the proper selection of a coating system is critical for the durability and reliability of the mould's working components. The comprehensive analysis of mechanical and tribological properties, as well as corrosion resistance, presented in this paper allows for the identification of relationships between the type of coating and the substrate material, which can serve as practical guidelines for selecting protective coatings based on the operating conditions of the tools. These results may help increase the durability of injection moulds, reduce wear on moving parts and improve the stability of manufacturing processes in the plastics industry. To the best of the authors' knowledge, no previous study has comparatively evaluated the mechanical, optical, tribological and hydrothermal corrosion performance of BALINIT and BALITHERM coating systems applied to four industrial mould steels (1.2343, 1.2083, 1.2378 and Toolox 44) under identical experimental conditions. This study addresses that gap and provides a comprehensive evidence-based framework for coating selection in polymer injection mould applications [32].

## 2. Material and methods

Four steel grades commonly used in the tool industry were selected for testing: 1.2343, 1.2083, 1.2378 and Toolox 44. Selected coating systems from the BALINIT and BALITHERM groups, that is, BALINIT LUMENA, BALINIT CROVEGA, BALINIT CROMA PLUS and BALITHERM PRIMEFORM, were applied to the prepared substrates. The steel grades and coatings used are listed in table 1.

**Table 1.** Materials used for testing, types of steel and coatings applied.

coating	BALINIT® LUMENA	BALINIT® CROVEGA	BALINIT® CROMA PLUS	BALITHERM® PRIMEFORM
steel grade	1.2343 ∼ 50 Rockwell C scale (HRC)			
	1.2083 ∼ 50 HRC				
	1.2378HH ∼ 36 HRC				
	Toolox 44 ∼ 45 HRC				

### 2.1. Physical vapour deposition process

**Figure 1.**
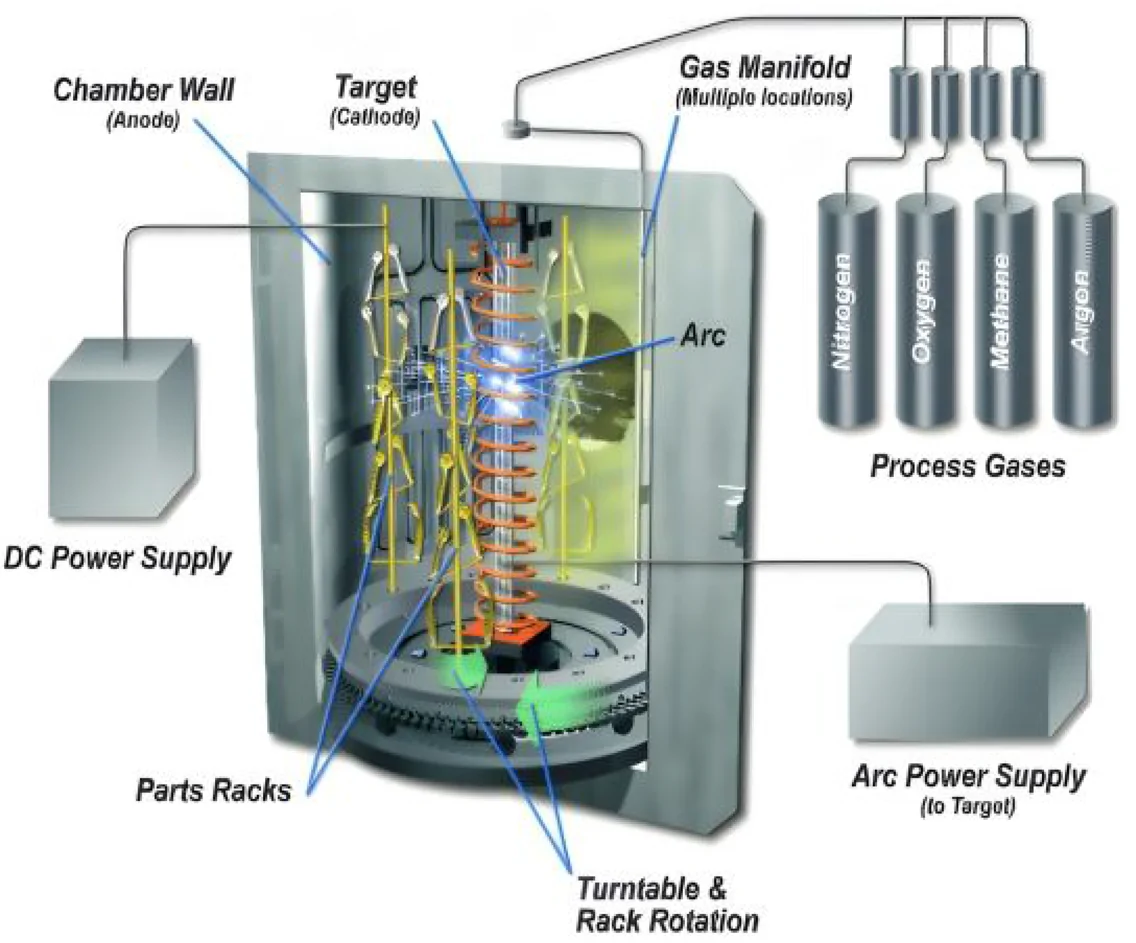
Diagram of a PVD chamber for applying thin coatings [33].

The conducted research utilized coatings based on TiAlN, CrN and the PRIMEFORM diffusion coating. Two PVD coating application methods were used: sputtering and arc evaporation. The common feature of both processes is that they occur at elevated temperatures between 250°C and 500°C in a high vacuum. In this process, a high-purity solid coating material (e.g. titanium, chromium or aluminium) is vaporized by heating or ion bombardment (sputtering). At the same time, a reactive gas (e.g. nitrogen) is added, forming a mixture with the metal vapours (figure 1). The resulting layer ultimately deposits on the components as a thin, strongly adherent coating. Typical coating thicknesses are 3–10 µm.

### 2.2. Scanning electron microscopy with elemental composition analysis (SEM–EDS)

Scanning electron microscopy (SEM) was used to observe metal samples, both reference samples and those with coatings. EDS analysis was used to confirm the composition of the metal samples and the coatings applied to them. Just before measurement, the samples were coated with a thin layer of gold using the sputtering method. This process was carried out using an automatic vacuum sputtering device, Smart Coater DII-29030SCTR (JEOL Ltd., Peabody, USA). Observations were made using a JEOL IT200 scanning microscope (JEOL Ltd., Peabody, MA, USA).

### 2.3. Optical microscopy

Morphological analysis was performed, including surface roughness of bare metal materials and materials with coatings applied. The structure of the materials was analysed using a Keyence VHX-7000 optical microscope (Keyence, Osaka, Japan). Based on three-dimensional (3D) surface measurements, profiles were determined, including linear roughness parameters: Ra (average roughness), Rq (root mean square deviation) and Rsk (skewness). Statistical analysis was carried out using one-way analysis of variance (ANOVA) to assess the effect of coating type for each steel grade. Post hoc comparisons were performed using Tukey's honestly significant difference (HSD) test to determine pairwise differences between coatings. The HSD values were calculated based on the mean square within groups, the number of observations and the studentized range distribution (*α* = 0.05). A difference was considered statistically significant when the absolute difference between group means exceeded the HSD value. The results are presented using letter-based groupings to indicate statistical significance.

### 2.4. The Vickers microhardness of the samples

Microhardness is an indicator of the resistance of metallic materials to plastic deformation. Measurements were performed using the Vickers method on steel samples, both uncoated and coated. The analysis aimed to determine the changes in hardness resulting from surface modification through the application of the tested coatings. Microhardness measurements were performed at a load of 1 kg and a magnification of 40×. Note that at this load level, the indentation depth exceeds the coating thickness (3–10 µm for PVD systems), and the measured values therefore represent the composite hardness of the coating–substrate system rather than the isolated coating hardness. This approach was intentionally selected to characterize the effective mechanical resistance of the entire coated surface under conditions representative of industrial mould operation. To complement these measurements, additional low-load hardness tests at HV0.1 were performed on selected samples; these are reported in table 3 and allow assessment of coating-level hardness with minimized substrate influence. A minimum of *n* = 3 indentations were performed per sample; results are reported as mean ± s.d. One-way ANOVA with Tukey's post hoc test (*α* = 0.05) was applied to assess the statistical significance of hardness differences between coating systems. The results are presented using letter-based groupings to indicate statistical significance [34,35].

**Table 2.** One-way ANOVA results for surface roughness (Ra).

steel grade	*F* (3,8)	*p*-value	significance
**1.2343**	46.33	2.11 × 10^−5^	***
**1.2083**	25.45	1.91 × 10^−4^	***
**1.2378**	2.82	0.107	ns
**Toolox 44**	30.46	9.99 × 10^−5^	***

***p < 0.001, ns—not significant.

**Table 3.** One-way ANOVA results for microhardness (HV1.0).

steel grade	*F* (4,10)	*p*-value	significance
**1.2343**	586.95	8.20 × 10^−12^	***
**1.2083**	766.53	2.17 × 10^−12^	***
**1.2378**	1453.68	8.94 × 10^−14^	***
**Toolox 44**	207.78	1.41 × 10^−9^	***

*** *p* < 0.001.

### 2.5. X-ray fluorescence

XRF chemical composition testing was performed on metal samples of 1.2378 steel coated with various coatings. The purpose of the analysis was to confirm the correct application of the layers and to verify their chemical composition. Each of the coatings applied to the steel substrate was tested separately. The measurements were performed using an S2 PUMA Series II energy-dispersive XRF spectrometer (Bruker, Billerica, MA, USA) at room temperature. The results obtained allowed the identification of characteristic elements in the coatings and were correlated with the results of elemental composition mapping performed on the same samples.

### 2.6. Tribological test

Tribological tests were conducted on four types of tool steel commonly used in moulds and tools for working at elevated temperatures: 1.2083, 1.2343, 1.2378 and Toolox 44. Each material was coated with one of two coatings applied by PVD: BALINIT LUMENA and BALINIT CROMA PLUS. A total of eight samples were prepared in the form of cuboids measuring 40 × 20 × 10 mm.

The tribological behaviour of the coatings was evaluated in a sliding friction test in a reciprocating system (ball-on-flat) using a tribometer test bench from Rtec Instruments. The counter sample was a 1/4-inch diameter bearing steel ball. The test parameters were as follows:

— normal load (F): 10 N,
— stroke length: 5 mm,
— number of cycles: 10 000,
— motion frequency: 5 Hz, and
— environment: laboratory air, temperature 21.3–21.5°C.

During the test, the friction force (*F_x_*) values were recorded on an ongoing basis, and the COF was calculated on their basis.

After completing the tests, the topography of the wear surface was analysed using optical interferometric profilometry (10× magnification lens). For each sample, three linear profiles were made across the wear mark, and then the average topography parameters were calculated in accordance with ISO 25178-2. In addition, 3D maps of the surface were generated to assess the morphology and volume of wear.

### 2.7. Ellipsometer

This article presents the optical analysis of a thin-film material (Croma plus coating) using VASE spectroscopy. Four samples made of different steel grades (1.2343, 1.2083, Toolox 44 and 1.2378) were measured. Based on measurements in the wavelength range from 192 to 1678 nm, the refractive index (*n*) and extinction coefficient (*k*) were determined. The results indicate strong dispersion in the UV range and stabilization of parameters in the visible and near-infrared ranges.

The measurements were performed using a VASE ellipsometer in the wavelength range from 192 to 1678 nm. The data were processed using a layered model, and the *n* and *k* coefficients were determined by model fitting to experimental data [36,37].

### 2.8. Glossmeter

Two measuring devices were used to determine the gloss values of individual samples. A 3Color® GM-38 glossmeter with Gloss QC software and a ZGM 1120 Glossmeter from Zehntner GmbH were used for the tests. Four samples were tested, each with a different coating. The coatings were applied to 1.2378HH steel. Measurements were taken for standard materials at an angle of 60° and for high-gloss surfaces at an angle of 20°. The goal was to determine the best top layer in terms of gloss, which is fundamental for injection moulding PC/PC-ABS materials into high-gloss products [38].

### 2.9. Corrosion test—hydrothermal incubation

Hydrothermal incubation was used to evaluate corrosion resistance under conditions representative of polymer injection moulds, where exposure to hot water vapour and condensate (60–120°C) is the primary degradation factor. Samples were immersed in distilled water and incubated at 80°C for 14 days. The pH and electrical conductivity of the solution were measured on days 1, 7 and 14 to monitor potential ion release using an Elmetron CX-701 pH-metre. Visual inspection of the samples was performed daily to detect signs of corrosion.

## 3. Results

### 3.1. Scanning electron microscopy with elemental composition analysis (SEM–EDS)

SEM images were taken of each of the samples produced at two magnifications, that is, ×500 and ×1000. EDS analysis and mapping were also performed. The results of EDS testing of uncoated samples confirmed the composition of the specific steels used to produce the samples. The exact elemental contents of the steel (1.2343, 1.2083, Toolox 44 and 1.2378) are included in the EDS report. EDS tests were also performed on the applied coatings. Each of the coatings (BALINIT LUMENA, BALINIT CROVEGA, BALINIT CROMA PLUS and BALITHERM PRIMEFORM) was applied to the four types of steel used. SEM images confirm the presence of coatings on the samples.

SEM image analysis allows for comparison of the surface topography of four tested steel grades in their uncoated state and after application of four different protective coatings. For each material, observations were made at magnifications of ×500 and ×1000, which enabled assessment of surface homogeneity and the quality of the applied layers.

It was observed that each type of coating takes on a similar appearance on different types of steel. EDS analysis was also performed on the coated samples and then compared to the composition of the applied coatings. This analysis confirmed the presence of coatings on the samples. EDS of some samples after coating showed the presence of iron Fe originating from steel. This is probably owing to the inaccurate application of the coating or its insufficient thickness. The increased carbon (C) content in the EDS analysis may be owing to the use of carbon pads for SEM testing. Mapping was also performed for each sample, which showed the distribution of the elements in a specific area of the sample.

#### 3.1.1. Steel 1.2343

The uncoated surface shows numerous signs of mechanical processing, clearly visible in both magnifications. After applying the BALINIT LUMENA coating, a more uniform structure is visible, with characteristic point defects. The BALINI CROVEGA coating gives a more uniform surface appearance, but minor irregularities are still visible. BALINIT CROMA PLUS shows the highest number of micropores and inclusions, especially at ×1000 magnification. BALITHERM PRIMEFORM, on the other hand, is characterized by a surface with distinct scratches, but at the same time relatively even coverage.

#### 3.1.2. Steel 1.2083

In its uncoated state, the surface is smooth with slight traces of machining. The application of BALINIT LUMENA results in the appearance of evenly distributed micropores. The BALINIT CROVEGA coating is characterized by a relatively smooth surface with fewer defects. BALINIT CROMA PLUS reveals a significant number of pores and inclusions, particularly visible at 1000× magnification. BALITHERM PRIMEFORM, on the other hand, is characterized by scratches and local surface irregularities.

#### 3.1.3. Steel 1.2378

The uncoated surface shows clear streaks and scratches after machining. BALINIT LUMENA forms a layer with scattered micropores, which appear irregular when magnified ×1000. BALINIT CROVEGA produces a more uniform surface, although with visible traces of the substrate. BALINIT CROMA PLUS is characterized by numerous pores and precipitates, indicating a less uniform deposition of the layer. BALITHERM PRIMEFORM retains mechanical traces of the substrate but forms a fairly regular coating.

#### 3.1.4. Toolox 44 steel

The uncoated sample has a relatively smooth surface with visible scratches. BALINIT LUMENA causes numerous pores to appear, distributed evenly. BALINIT CROVEGA produces a surface with fine scratches, but with increased smoothness compared to the substrate. BALINIT CROMA PLUS, as with other grades, is characterized by a large number of pores, particularly visible at 1000× magnification. BALITHERM PRIMEFORM shows clear signs of substrate treatment, but the coating covers them relatively evenly.

### 3.2. Mapping

In addition, EDS mapping was performed on uncoated samples (figures 2–9), which allowed the chemical composition of individual steel grades to be confirmed. This analysis was important for further evaluation, as it provided a reference to the actual composition of the substrate material.

**Figure 2.**
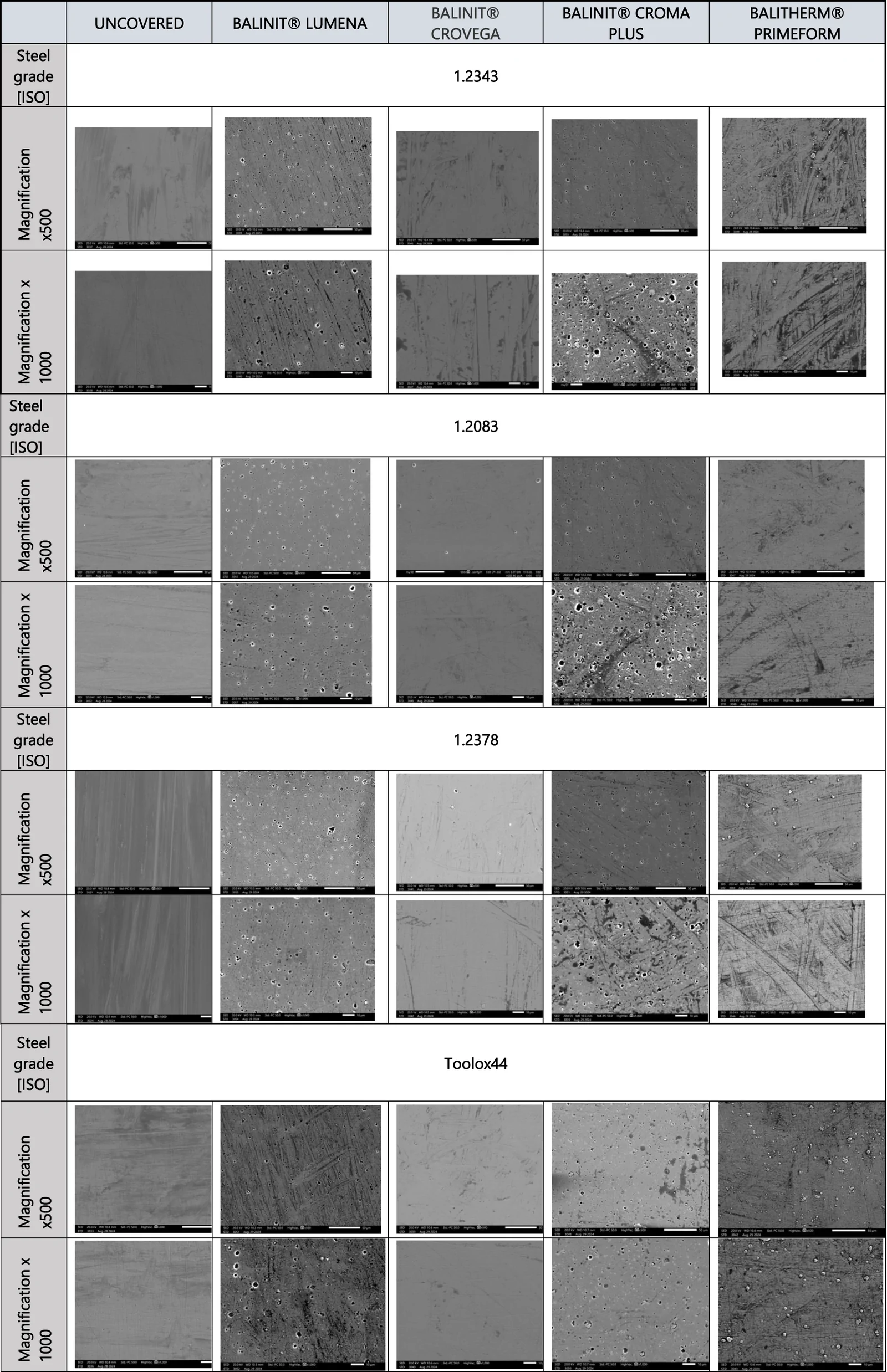
SEM analysis.

**Figure 3.**
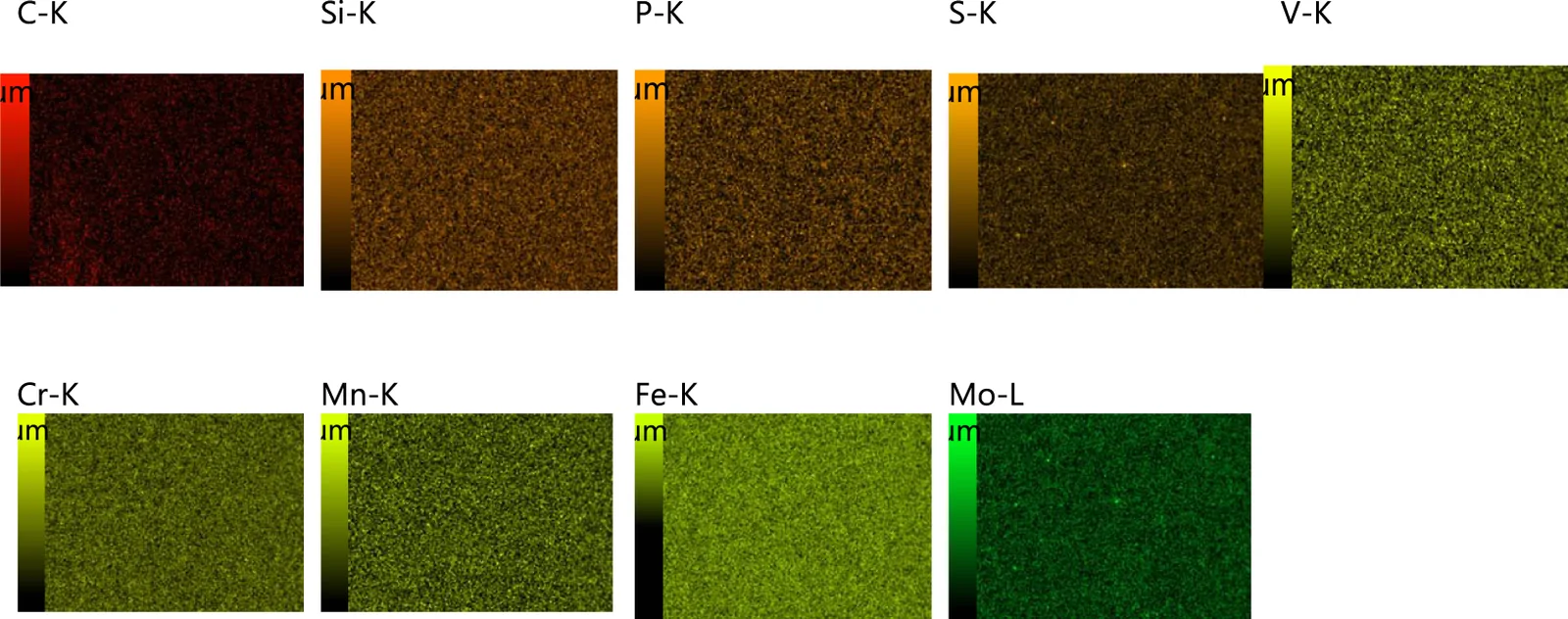
Element distribution map (mapping) of 1.2343 steel sample.

**Figure 4.**
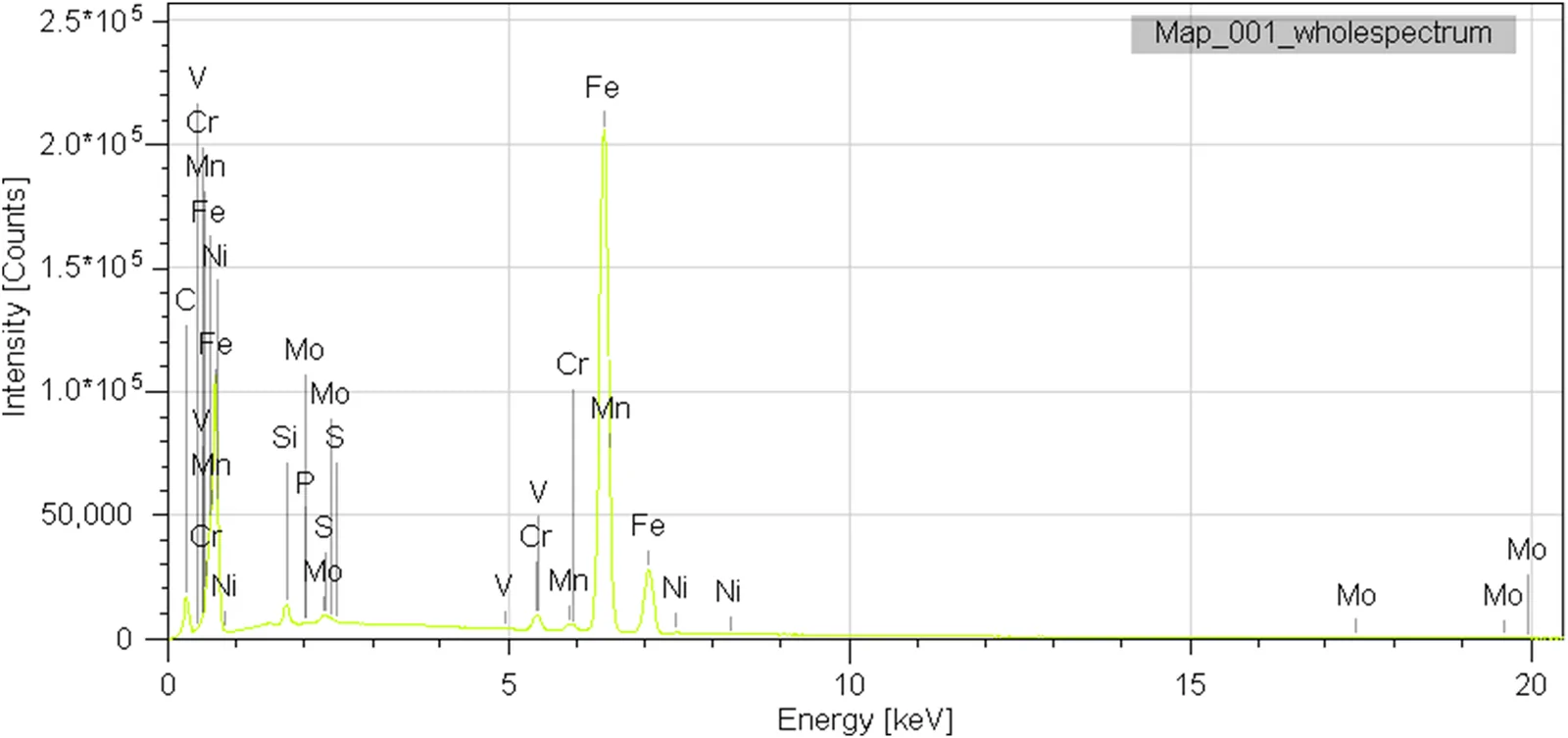
EDS spectrum showing the intensity of signals characteristic of elements present in the 1.2343 steel sample.

**Figure 5.**
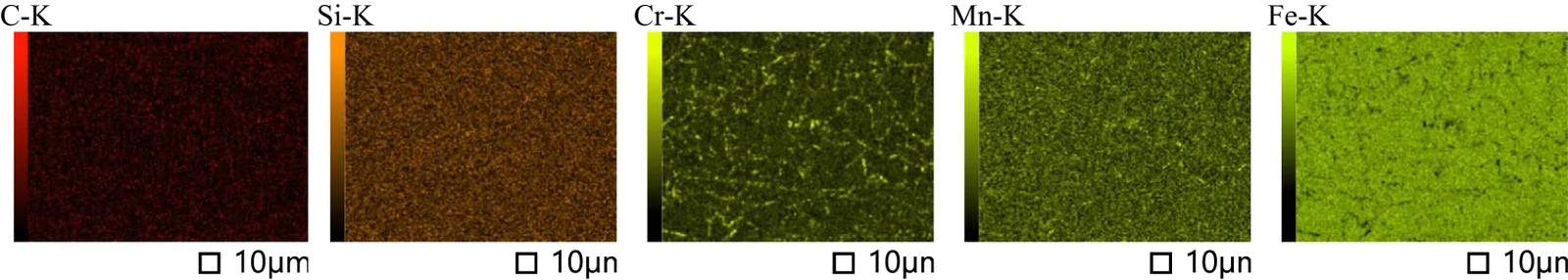
Element distribution map (mapping) of 1.2083 steel sample.

**Figure 6.**
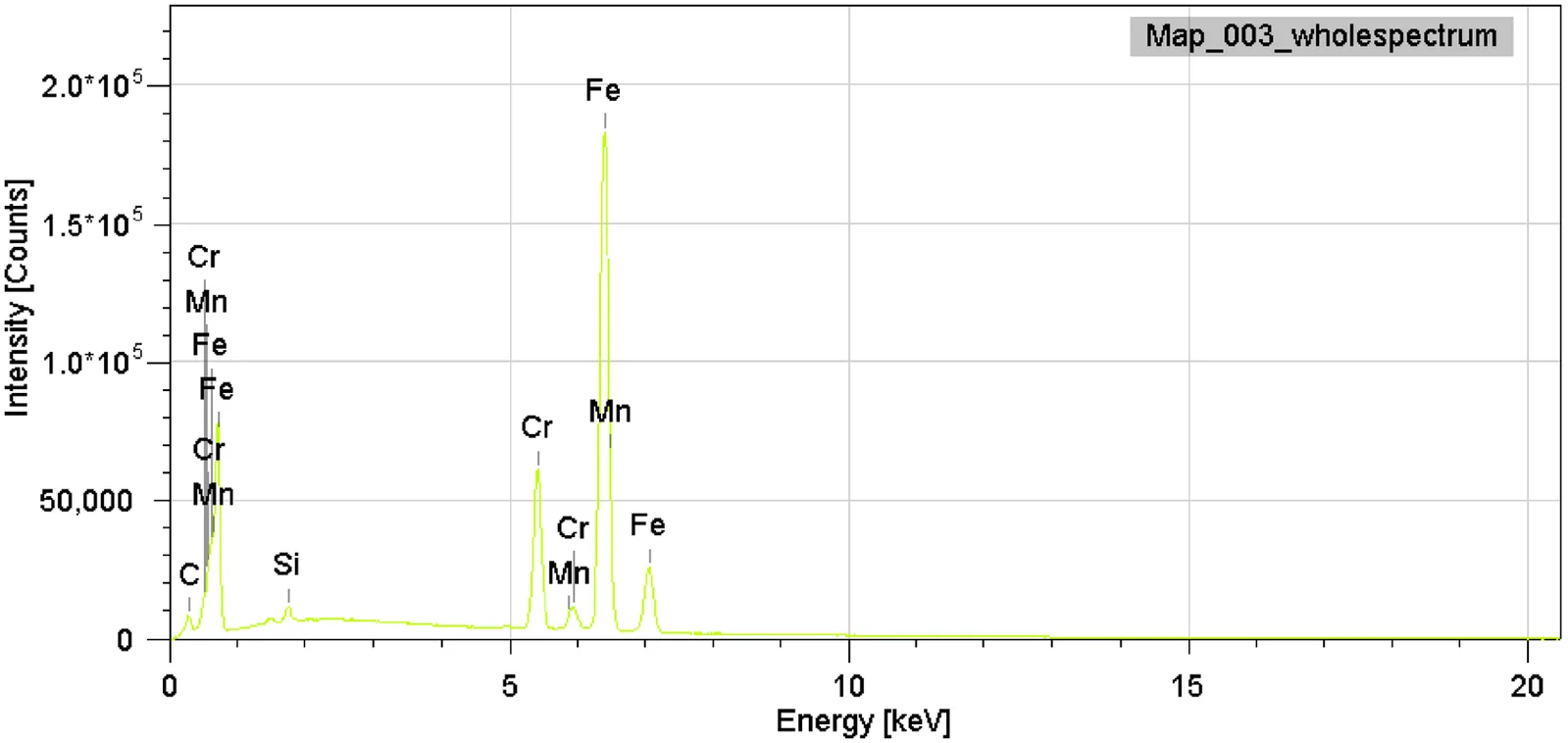
EDS spectrum showing the intensity of signals characteristic of elements present in the 1.2083 steel sample.

**Figure 7.**
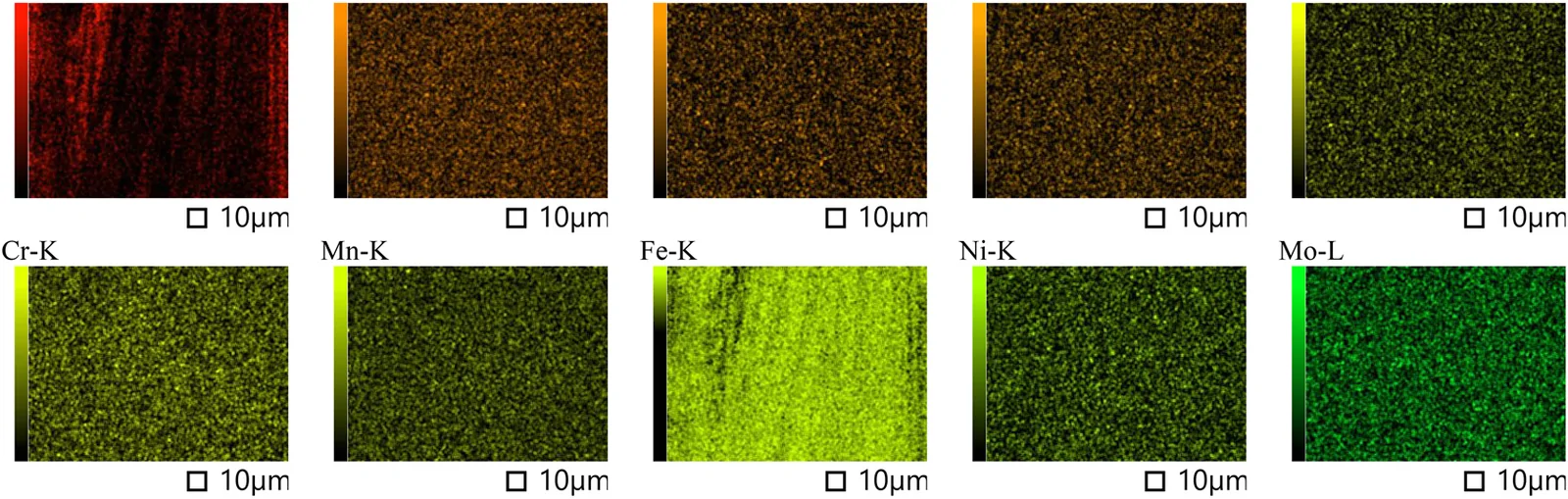
Element distribution map (mapping) of 1.2378 steel sample.

**Figure 8.**
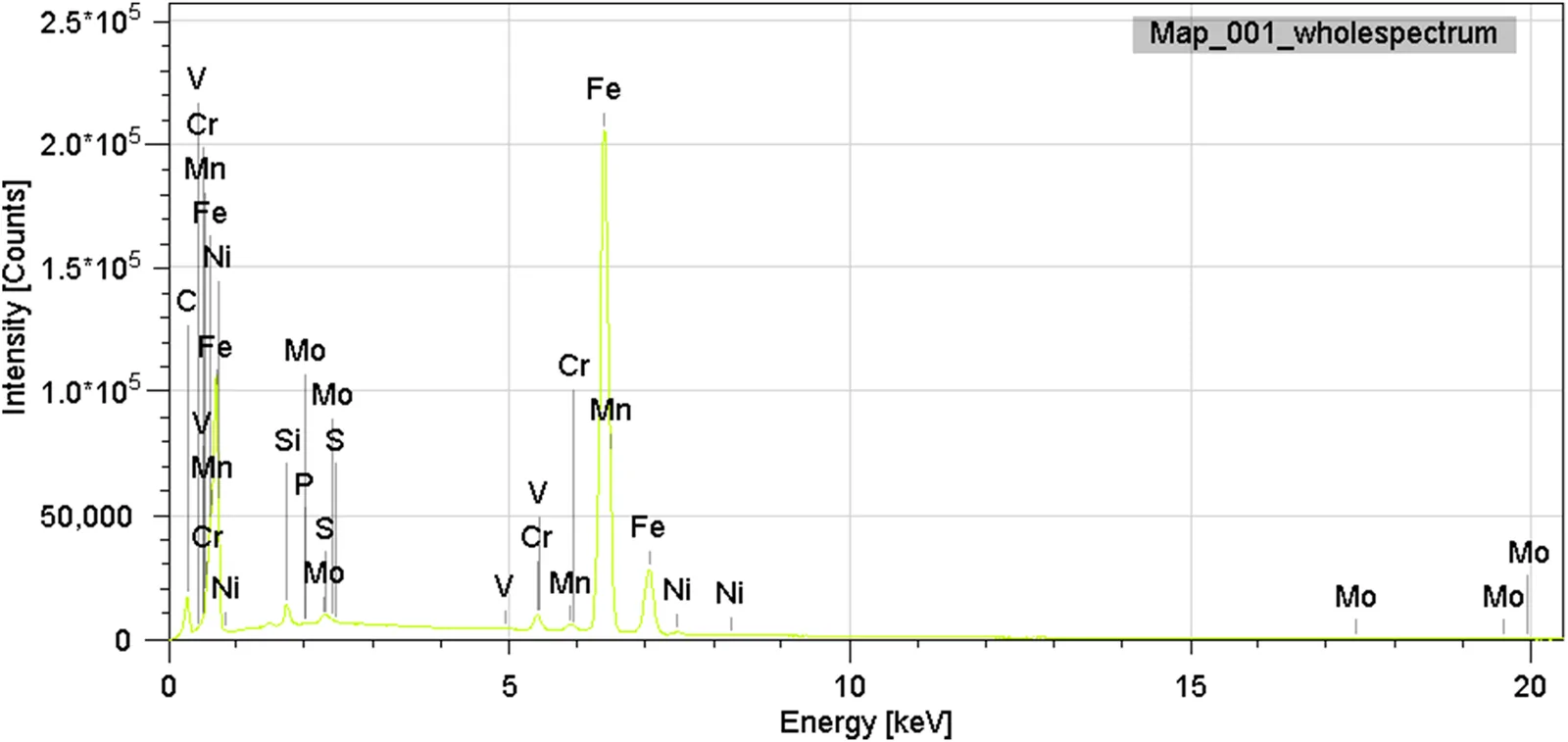
EDS spectrum showing the intensity of signals characteristic of elements present in the 1.2378 steel sample.

**Figure 9.**
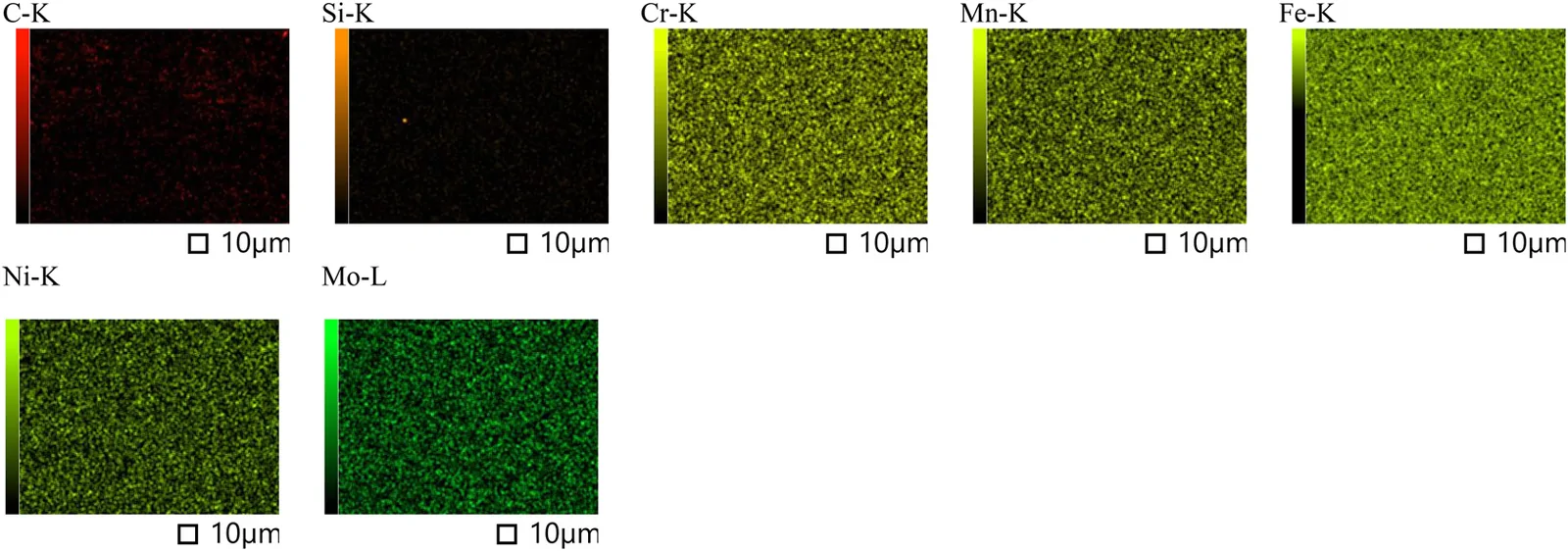
Element distribution map (mapping) of the Toolox 44 steel sample.

**Figure 10.**
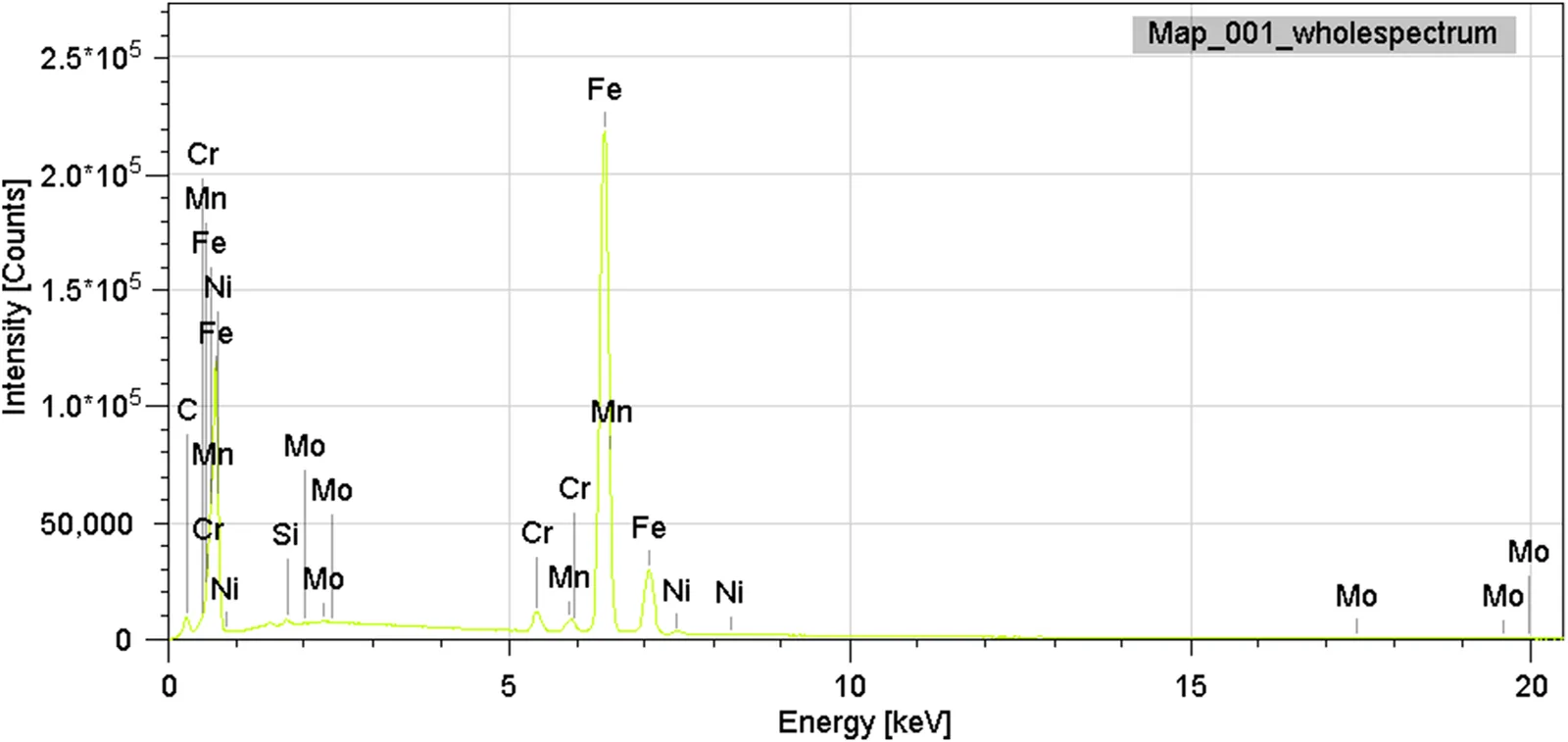
EDS spectrum showing the intensity of signals characteristic of elements present in the Toolox 44 steel sample.

**Figure 11.**
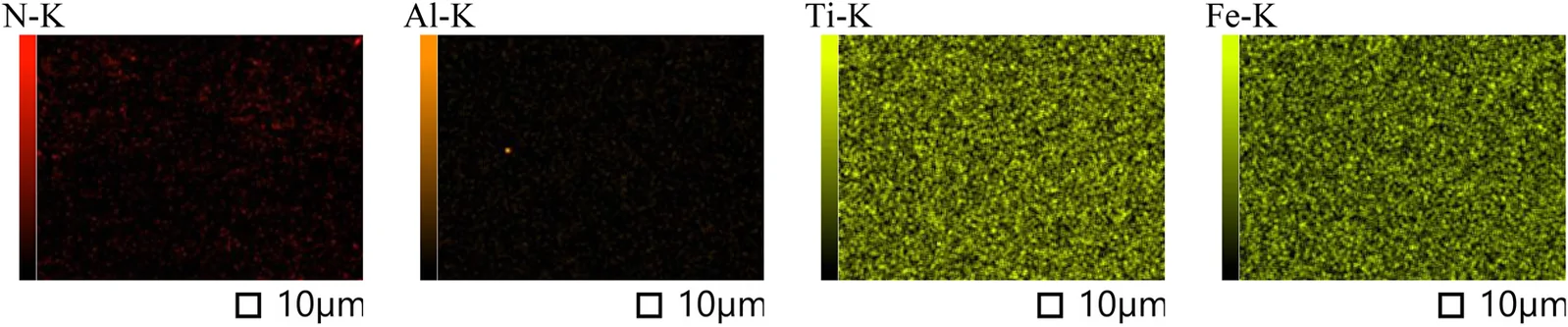
Element distribution map (mapping) of 1.2378 steel sample coated with BALINIT LUMENA.

**Figure 12.**
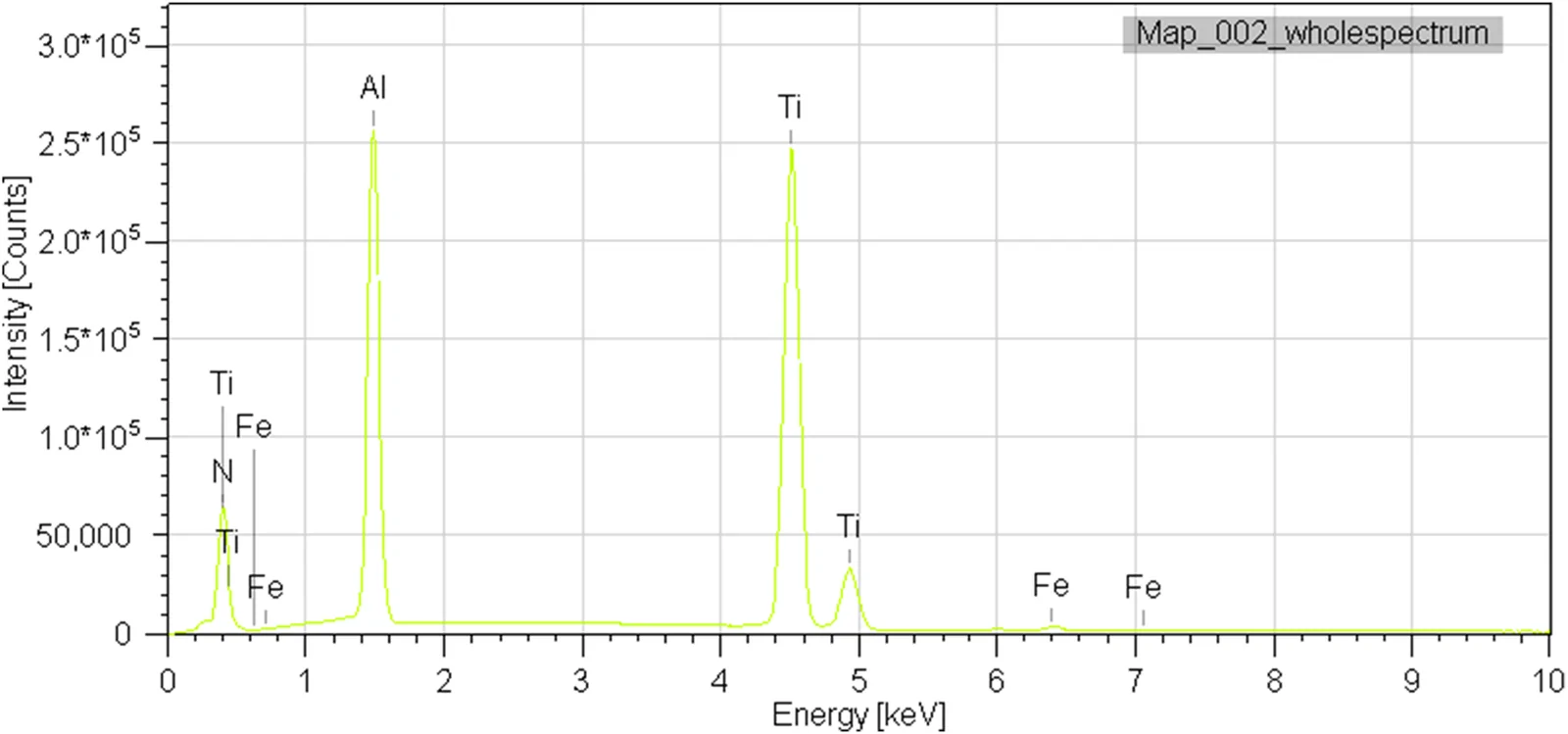
EDS spectrum showing the intensity of signals characteristic of elements present on the surface of a 1.2378 steel sample coated with BALINIT LUMENA.

**Figure 13.**
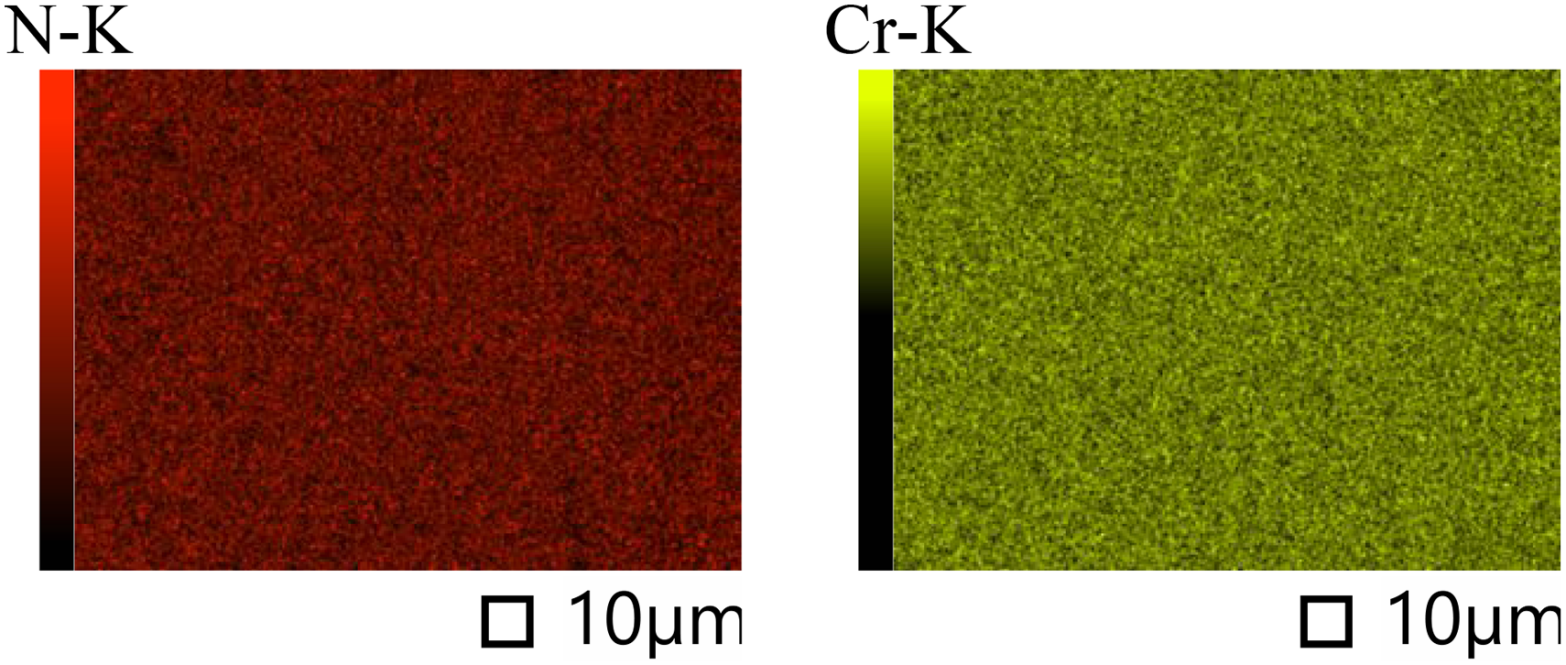
Element distribution map (mapping) of 1.2378 steel sample coated with BALINIT CROVEGA.

**Figure 14.**
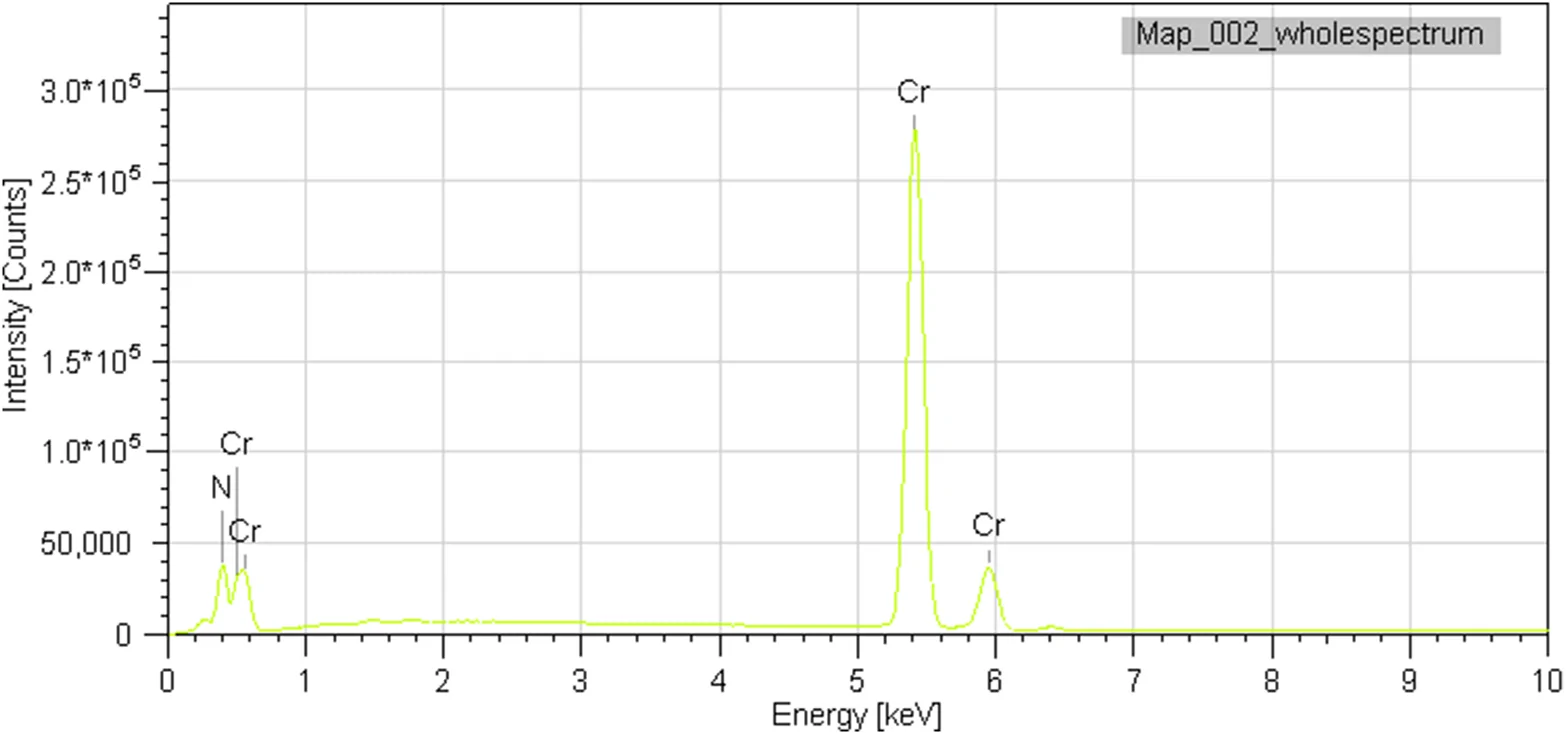
EDS spectrum showing the intensity of signals characteristic of elements present on the surface of a 1.2378 steel sample coated with BALINIT CROVEGA.

**Figure 15.**
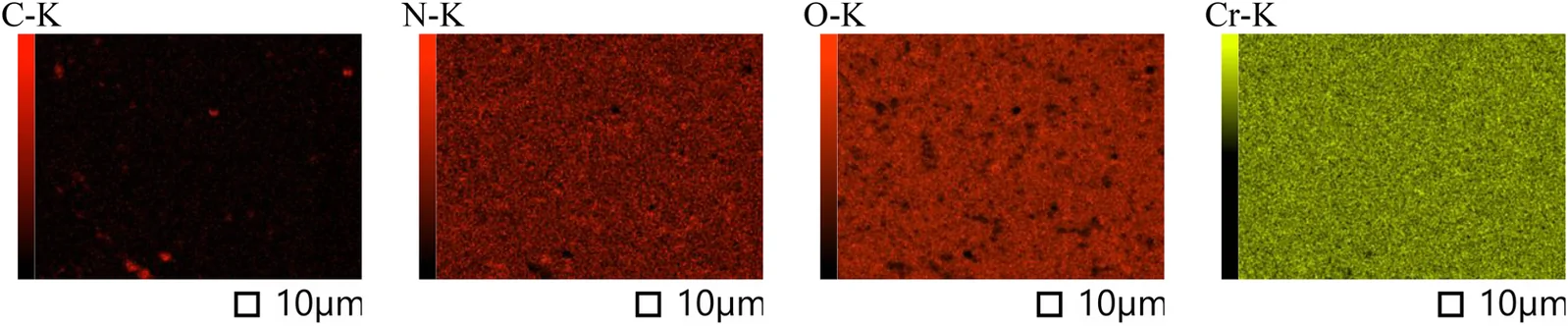
Element distribution map (mapping) of 1.2378 steel sample coated with BALINIT CROMA PLUS.

**Figure 16.**
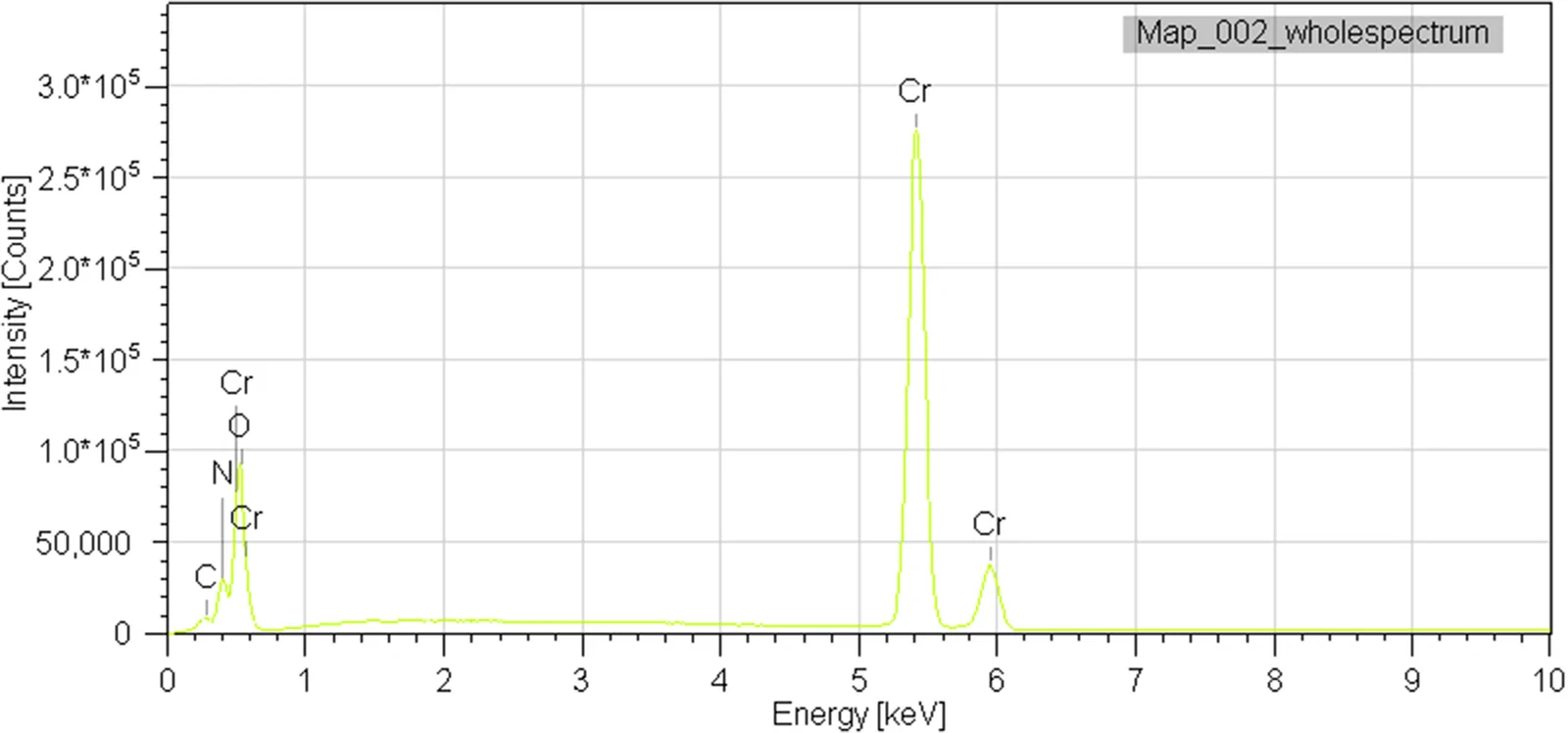
EDS spectrum showing the intensity of signals characteristic of elements present on the surface of a 1.2378 steel sample coated with BALINIT CROMA PLUS.

**Figure 17.**
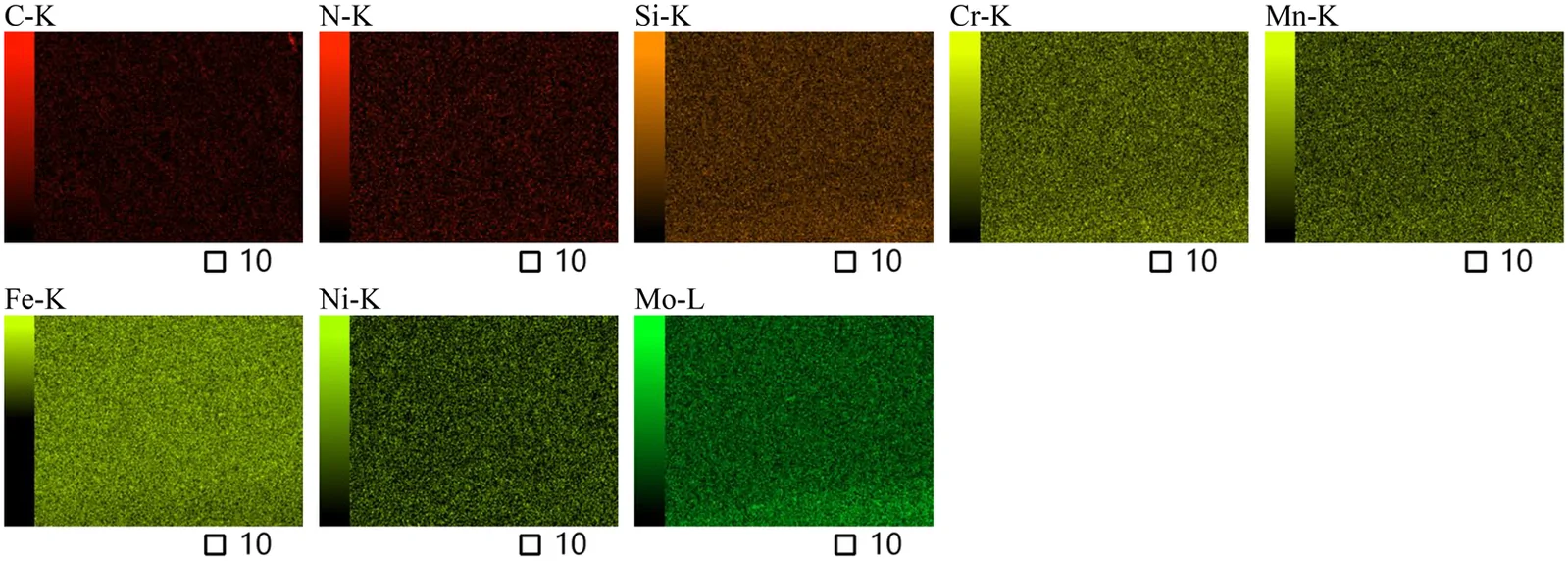
Element distribution map (mapping) of 1.2378 steel sample coated with BALITHERM PRIMEFORM.

**Figure 18.**
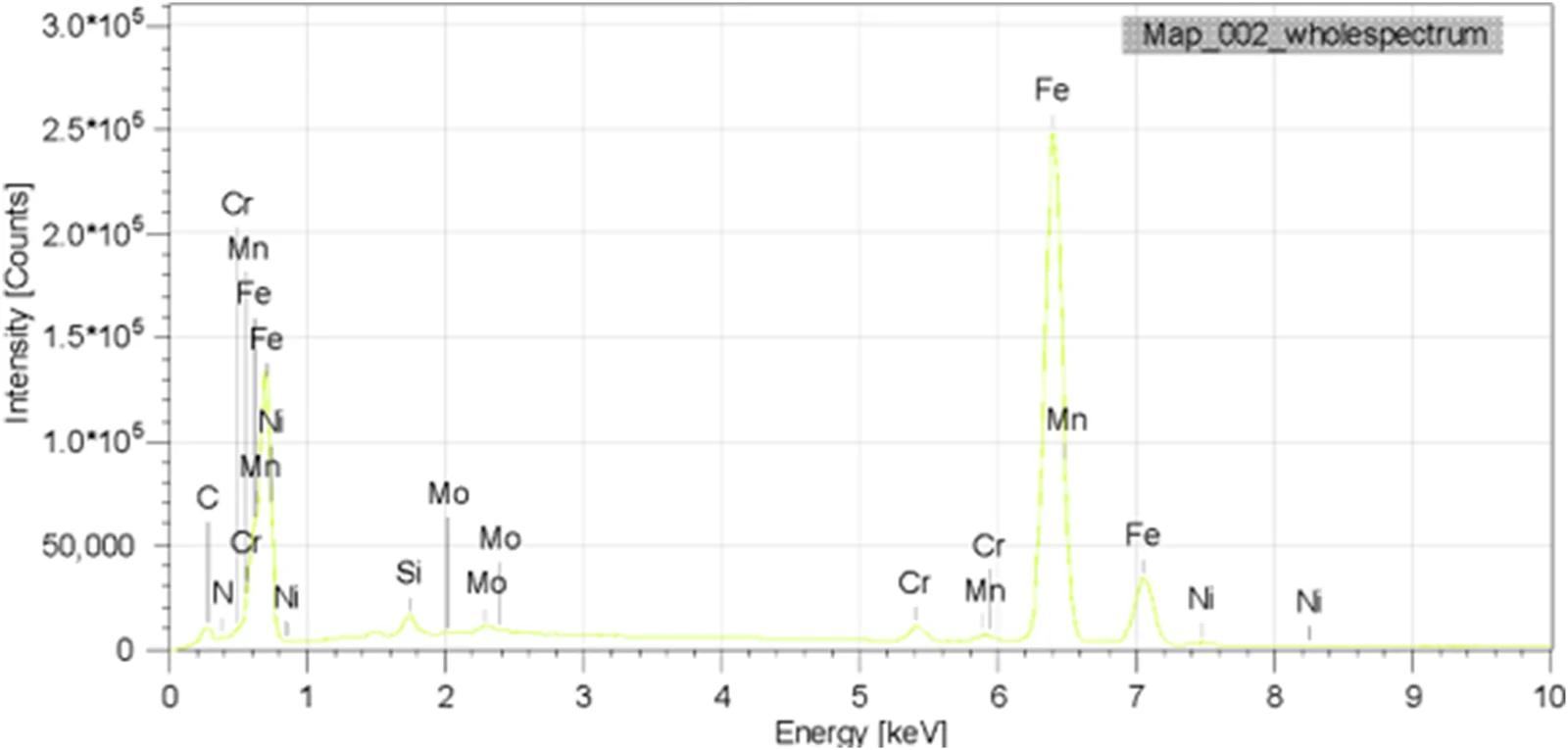
EDS spectrum showing the intensity of signals characteristic of elements present on the surface of a 1.2378 steel sample coated with BALITHERM PRIMEFORM.

**Figure 19.**
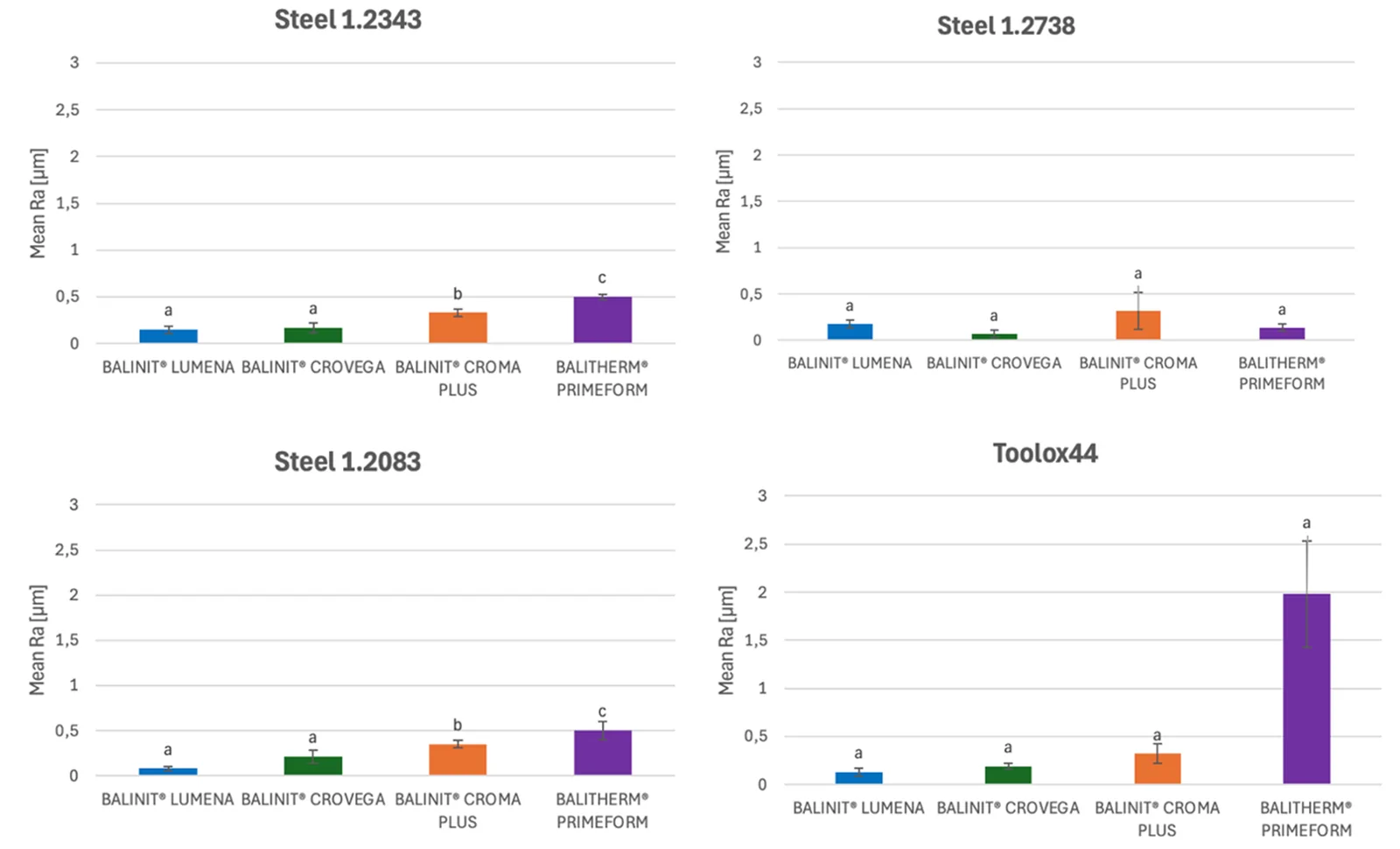
Surface roughness (Ra) for different coatings deposited on selected steel grades: 1.2343, 1.2083, 1.2378 and Toolox 44. Values are presented as mean ± s.d. (*n* = 3). Different letters indicate statistically significant differences between coatings according to Tukey's HSD test (*α* = 0.05).

Mapping was also performed for coated samples (figures 10–19), but it was limited to one steel grade—1.2378. The purpose of this analysis was to confirm the effectiveness of the coating application process and to verify its chemical composition. The results clearly indicated the presence of characteristic elements corresponding to the given types of coatings, which confirms their correct deposition on the substrate surface.

### 3.3. Optical microscopy and roughness

Analysis of the results of surface roughness measurements of PVD and CVD coatings applied to various types of steel showed a significant influence of both the type of coating and the substrate material on the obtained surface topography parameters. The differences in the arithmetic mean roughness Ra, which is the basic criterion for assessing surface quality in the context of industrial applications, were particularly noticeable. The results are shown in figure 19.

In the case of 1.2343 steel, commonly used in hot-work tools, the most favourable results were obtained for BALINIT LUMENA and BALINIT CROVEGA coatings, which provided Ra values of 0.15 and 0.17 µm, respectively. By contrast, the BALITHERM PRIMEFORM coating significantly deteriorated the surface quality (Ra = 0.50 µm), which may limit its usefulness in applications requiring high smoothness.

Stainless steel 1.2083 proved to be particularly sensitive to the type of coating. The lowest Ra value of 0.08 µm was recorded for the BALINIT LUMENA coating, indicating very good chemical and adhesive compatibility of this coating with the substrate. The Ra values for BALINIT CROMA PLUS and BALINIT CROVEGA were higher (0.21 and 0.35 µm, respectively), while the use of BALITHERM PRIMEFORM resulted in a significant increase in roughness (Ra = 0.50 µm).

The best results were obtained for steel 1.2378, where all analysed coatings maintained very low Ra values. The lowest surface roughness was recorded for BALINIT CROVEGA (Ra = 0.07 µm), and the other coatings also provided a favourable level of smoothness (Ra in the range of 0.14–0.32 µm). This proves the high susceptibility of this steel to coating application while maintaining a uniform topography.

By contrast, the heat-treated Toolox 44 steel showed high variability in the parameters obtained depending on the coating. The best smoothing effect was achieved with BALINIT LUMENA (Ra = 0.13 µm). By contrast, the BALITHERM PRIMEFORM coating caused a significant deterioration in surface quality (Ra = 1.98 µm), which completely disqualifies this material-coating combination for applications requiring precise surface reproduction.

One-way ANOVA was performed to evaluate the effect of coating type on surface roughness (Ra) for each investigated steel grade (table 2). The analysis revealed statistically significant differences between coatings for steels 1.2343**,** 1.2083 and Toolox 44 (*p* < 0.001). In these cases, the calculated *F*-values (46.33, 25.45 and 30.46, respectively) were substantially higher than the critical *F* value (*F*_crit_ ≈ 4.07), indicating that the variability between coating groups greatly exceeded the variability within groups.

By contrast, for steel 1.2378, no statistically significant differences were observed (*F* = 2.82, *p* = 0.107), suggesting that the coating type does not significantly influence the Ra parameter for this material.

A post hoc Tukey's HSD test was performed to identify statistically homogeneous groups of coatings for each steel grade (figure 19). For steels 1.2343, 1.2378 and Toolox 44, the coatings were divided into distinct homogeneous groups, indicating significant differences between selected coatings. BALITHERM® PRIMEFORM consistently formed a separate group with the highest Ra values, clearly differing from the other coatings.

By contrast, coatings such as BALINIT® LUMENA and BALINIT® CROVEGA frequently belonged to the same homogeneous group, indicating no statistically significant differences between them. For steel 1.2083, the grouping was less distinct, with partial overlap between coatings, confirming the lack of statistically significant differences observed in the ANOVA.

Surface roughness tests showed that the base steel grade has a significant influence on the quality of the BALINIT CROMA PLUS coating. Ra values ranged from 0.09 to 0.31 µm, demonstrating a clear variation in results depending on the substrate.

For hot-work tool steel (1.2343), Ra = 0.28 µm was obtained. The 3D map and linear profile (figure 19) revealed moderate amplitude irregularities, distributed evenly across the entire surface. The coating settled stably but did not provide perfect smoothness—the effect is correct but not optimal compared to other steels.

**Figure 20.**
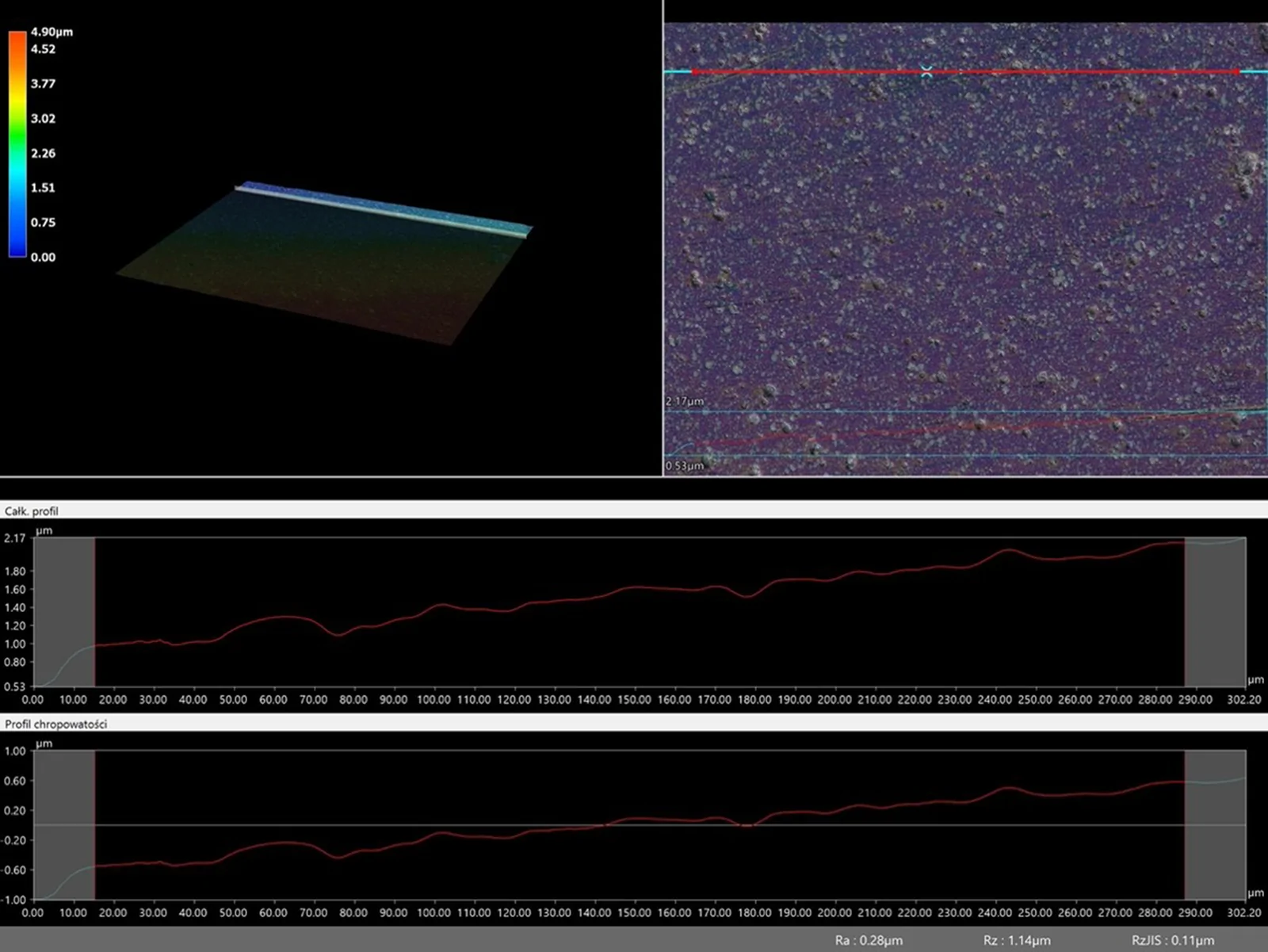
Microscopic image and corresponding 3D surface profiles of BALINIT CROMA PLUS on steel 1.2343.

On stainless steel for moulds (1.2083), roughness increased to Ra = 0.31 µm and Rz = 1.57 µm (figure 20). The surface profile indicates the presence of ridges and valleys associated with the microstructure of the steel, which resulted in a significantly greater surface unevenness of the coating. In this case, BALINIT CROMA PLUS does not provide smoothness comparable to other coatings, such as BALINIT LUMENA.

**Figure 21.**
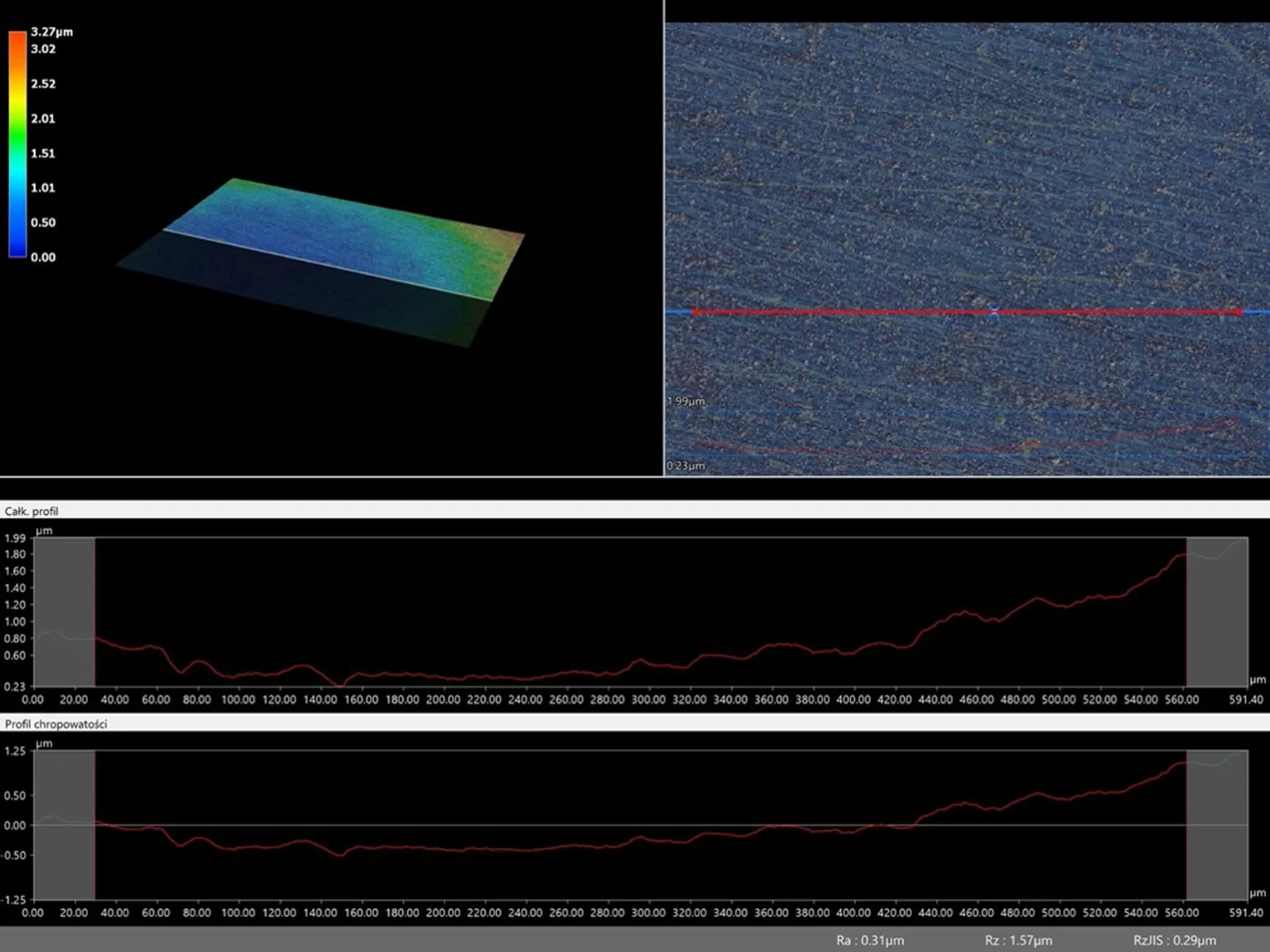
Microscopic image and corresponding 3D surface profiles of BALINIT CROMA PLUS on steel 1.2083.

The best results were obtained for mould steel 1.2378—Ra = 0.09 µm and Rz = 0.71 µm. The roughness profile and 3D map (figure 21) showed an exceptionally even and homogeneous surface with minimal height differences. The coating spread very evenly, and the existing traces of mechanical processing did not adversely affect the final smoothness level. This combination shows the highest compatibility between the substrate and the coating.

**Figure 22.**
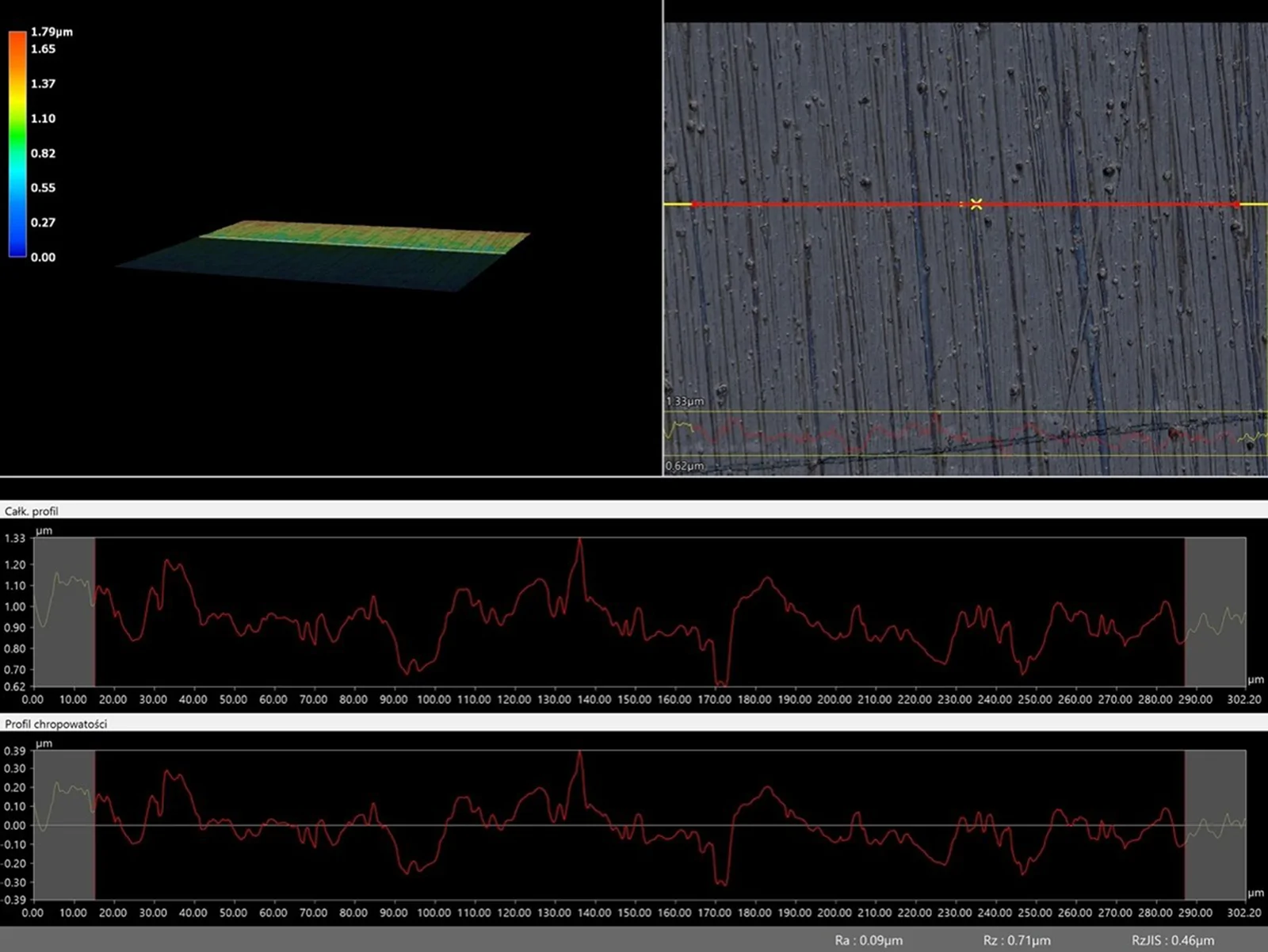
Microscopic image and corresponding 3D surface profiles of BALINIT CROMA PLUS on steel 1.2378.

**Figure 23.**
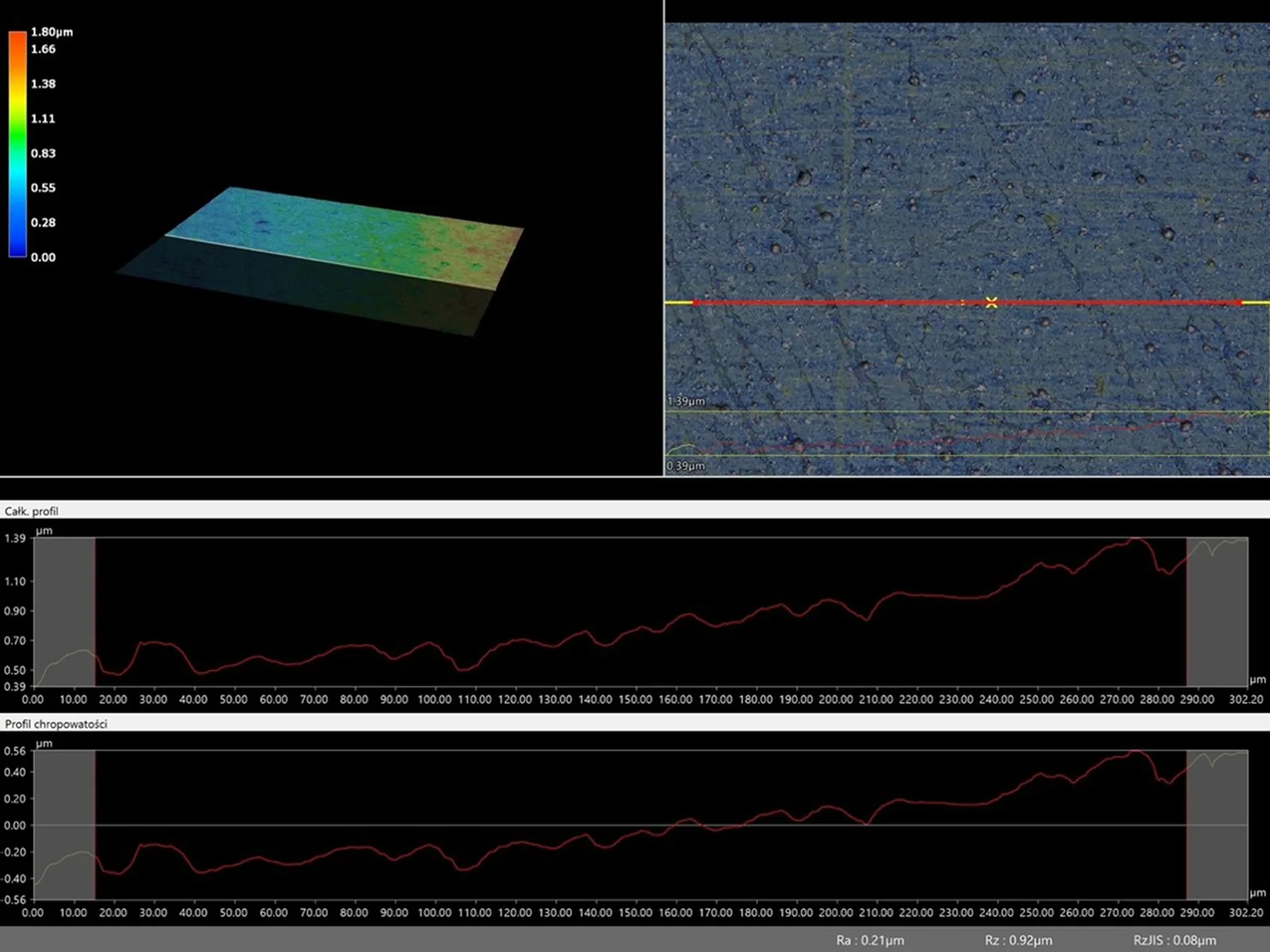
Microscopic image and corresponding 3D surface profiles of BALINIT CROMA PLUS on steel Toolox 44.

In the case of heat-treated Toolox 44 steel, intermediate values were obtained—Ra = 0.21 µm, Rz = 0.92 µm (figure 23). The 3D map shows (figure 22) point irregularities and local inclusions, which cause a slight but noticeable increase in roughness. Although the result is better than for 1.2083 steel, it does not match the smoothness of 1.2378.

**Figure 24.**
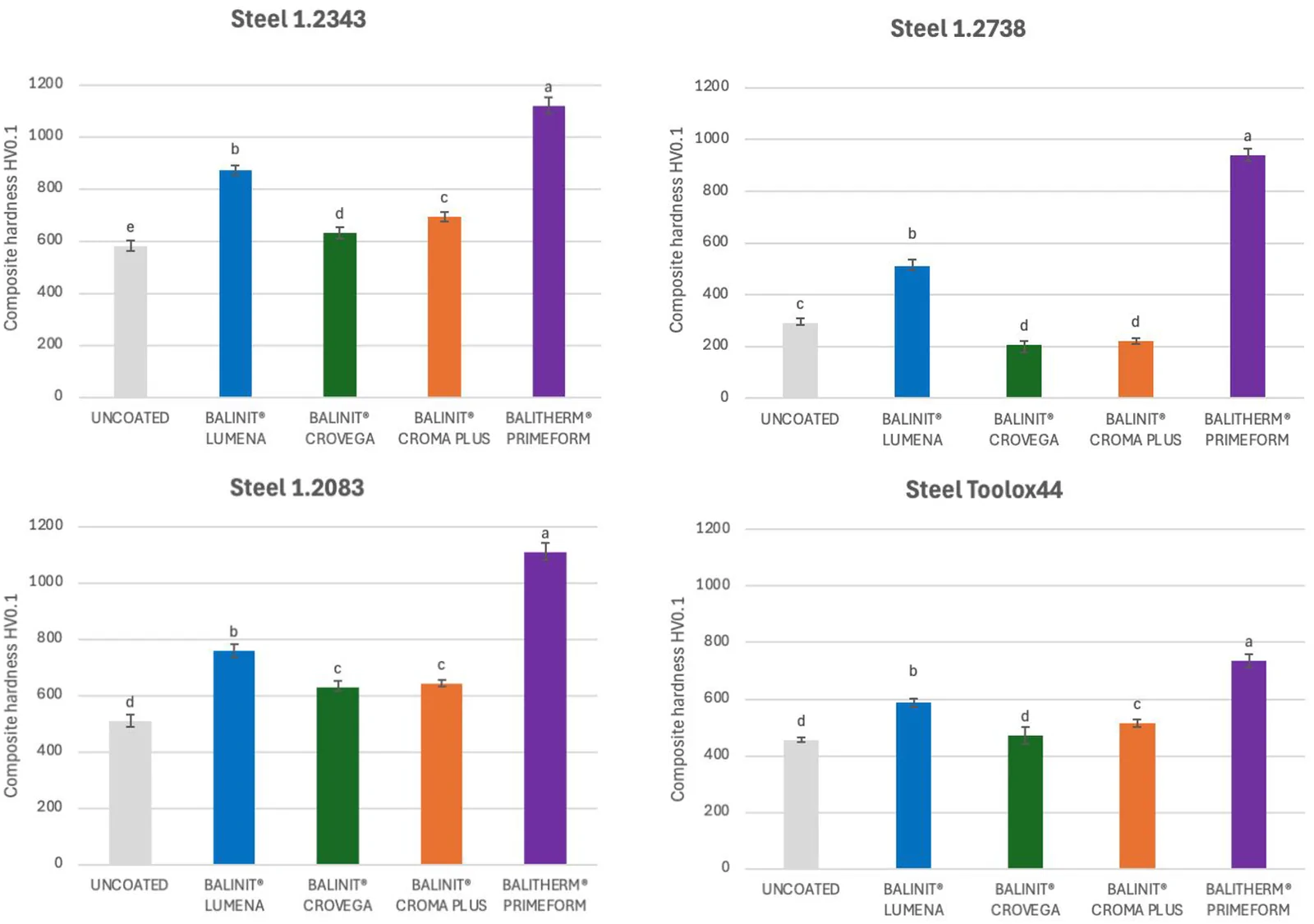
Vickers hardness (HV1.0) for different coatings deposited on selected steel grades: 1.2343, 1.2083, 1.2378 and Toolox 44. Values are presented as mean ± s.d. (*n* = 3). Different letters indicate statistically significant differences between coatings according to Tukey's HSD test (*α* = 0.05).

### 3.4. The Vicker microhardness results

In order to determine the effect of the type of protective coating on the mechanical properties of the substrate, microhardness measurements were performed using the Vickers method (HV1) for four variants of commercial coatings applied to different types of steel (figure 24).

#### 3.4.1. Vickers microhardness testing for coatings on steel 1.2343

Here we describe the process applied to hot-work tool steel grade 1.2343 (X37CrMoV5-1).

The initial condition of the material (without coating) was characterized by an average microhardness of 582 HV1 (measurement values: 573, 592, 582 HV1), which serves as a reference point for evaluating the effect of surface modification. This result corresponds to the typical value for 1.2343 steel after heat treatment (hardening and tempering), ensuring a compromise between hardness and fracture resistance.

The use of coatings led to a significant variation in surface hardness, which clearly indicates the significant influence of the type of coating on the mechanical properties of the surface layer. The highest microhardness was observed for samples treated with BALITHERM PRIMEFORM, for which the average value was 1119 HV1, which represents an increase of approximately 92% compared to the uncoated material. Such high hardness is the result of the diffusion nature of the process, leading to the saturation of the surface layer with stiffening elements (e.g. nitrogen, carbon), which increases resistance to plastic deformation and abrasion. The second most effective coating was BALINIT® LUMENA (TiAlN), which achieved an average microhardness of 871 HV1, corresponding to an increase of approximately 49% compared to the substrate. In the case of BALINIT® CROMA PLUS (CrN) and BALINIT® CROVEGA (CrN/TiN) coatings, a moderate increase in microhardness was recorded—692 HV1 (an increase of 19%) and 632 HV1 (an increase of 9%), respectively.

These results indicate that TiAlN coatings and diffusion processes are most effective in improving mechanical properties, while coatings based on chromium nitrides primarily serve a tribological and corrosion protection function, providing moderate hardness improvement while maintaining favourable surface properties.

#### 3.4.2. Vickers microhardness testing for coatings on steel 1.2083

In the Vickers microhardness tests (HV1) conducted on 1.2083 (X40Cr14) steel in its initial state, an average value of 509 HV1 was obtained, which serves as a reference point for assessing the effect of protective coatings. The use of PVD and CVD coatings resulted in a significant increase in surface hardness, with the values observed varying depending on the type of coating. The highest microhardness was achieved for samples after the BALITHERM® PRIMEFORM process (1111 HV1), which is more than twice that of the uncoated material. The results confirm the high effectiveness of diffusion processes in modifying the surface layer of steel, leading to its saturation with stiffening elements, which significantly increases resistance to plastic deformation and abrasion. A similar trend, albeit less intense, was observed for the BALINIT® LUMENA (TiAlN) coating, whose average microhardness was 759 HV1, corresponding to an increase of approximately 49% compared to the substrate. The BALINIT® CROVEGA and BALINIT® CROMA PLUS coatings showed a smaller increase in hardness (629 HV1 and 642 HV1, respectively), indicating that their main role is to improve tribological properties and corrosion resistance rather than to significantly increase hardness. The test results confirm that the choice of coating technology for 1.2083 steel should be closely related to the application requirements—diffusion processes are recommended for applications with critical wear resistance, while PVD coatings, especially TiAlN, are more advantageous in cases requiring low roughness and precise detail reproduction.

#### 3.4.3. Vickers microhardness testing for coatings on steel 1.2378

In microhardness tests using the Vickers method (HV1) for 1.2378 steel in its initial state, an average value of 291 HV1 (range: 278–297 HV1) was obtained, which is typical for this type of steel in a heat-treated state. The use of protective coatings revealed clear differences in the effectiveness of individual technologies. The highest values were obtained for samples subjected to the BALITHERM PRIMEFORM process, for which the average microhardness was 938 HV1 (range: 936–941 HV1), which corresponds to a more than threefold increase compared to the uncoated material. Such a significant increase in hardness results from the diffusive nature of the process, leading to a deep modification of the steel surface layer, increasing its resistance to abrasion and plastic deformation. A significant increase was also recorded for the BALINIT LUMENA (TiAlN) coating, whose average microhardness reached 512 HV1, which corresponds to an improvement of approximately 76% compared to the initial state. In the case of BALINIT CROVEGA and BALINIT CROMA PLUS coatings, the hardness values obtained were lower than for the substrate—207 HV1 and 223 HV1, respectively—which may be owing to the limited adhesion of these coatings to 1.2378 steel or different mechanisms of chromium nitride layer deposition, resulting in a reduction in the effective mechanical resistance of the surface. These results clearly indicate that for 1.2378 steel, the most effective technology for increasing surface microhardness is the BALITHERM PRIMEFORM process, while TiAlN-type PVD coatings may be a favourable solution in applications requiring moderate mechanical reinforcement while maintaining good tribological stability.

#### 3.4.4. Vickers microhardness testing for coatings on steel Toolox 44

In microhardness tests using the Vickers method (HV1) for Toolox 44 steel in its initial state, an average value of 457 HV1 was obtained (range: 454–460 HV1), which corresponds to the typical properties of this material supplied in a heat-treated state, combining high hardness with good impact resistance. The use of protective coatings had a varied effect on the mechanical properties of the surface layer. The highest values were recorded for samples treated with BALITHERM PRIMEFORM, for which the average microhardness was 735 HV1 (range: 719–754 HV1), representing an increase of approximately 61% compared to the uncoated material. A significant increase was also recorded for the BALINIT LUMENA (TiAlN) coating, reaching an average of 587 HV1, which represents an increase of 28% compared to the substrate, while BALINIT CROMA PLUS (CrN) showed a moderate increase to 515 HV1 (an increase of 13%). A different effect was observed for the BALINIT CROVEGA coating, whose average microhardness was 472 HV1, only slightly above the substrate value. These results indicate that for Toolox 44 steel, the most effective process for improving resistance to plastic deformation and abrasion is BALITHERM PRIMEFORM, this technology is the most effective process for improving resistance to plastic deformation and abrasion, while PVD coatings provide only moderate reinforcement, with the TiAlN coating (BALINIT LUMENA) showing significantly greater effectiveness than coatings based on chromium nitrides (CROVEGA, CROMA PLUS).

The ANOVA results confirm a highly significant effect of coating type on composite HV1 for all four steel grades (table 3; *F*(4,10) ranging from 208 to 1454, *p* < 0.001 in all cases). Tukey's HSD identifies a consistent hardness hierarchy across steels: PRIMEFORM (group a) > LUMENA (group b) > CROMA PLUS and CROVEGA (group c) > uncoated (group d/e). Key findings: (i) PRIMEFORM achieves the highest composite hardness (735 ± 22 to 1119 ± 31 HV1), significantly exceeding all PVD systems (*p* < 0.001 in all pairwise comparisons); (ii) LUMENA provides a statistically significant hardness improvement over all CrN-based coatings on every substrate tested; (iii) CROMA PLUS and CROVEGA are not statistically different from each other on any substrate (*p* > 0.05 in all cases), indicating equivalent composite mechanical performance despite their distinct chemical compositions; (iv) On steel 1.2378, CROVEGA (207 ± 14 HV1) and CROMA PLUS (223 ± 9 HV1) are not significantly different from either each other or the uncoated substrate (291 ± 19 HV1; *p* > 0.05), confirming that HV1 measurements on this softer steel (approx. 36 HRC) do not resolve coating-level hardness.

**Figure 25.**
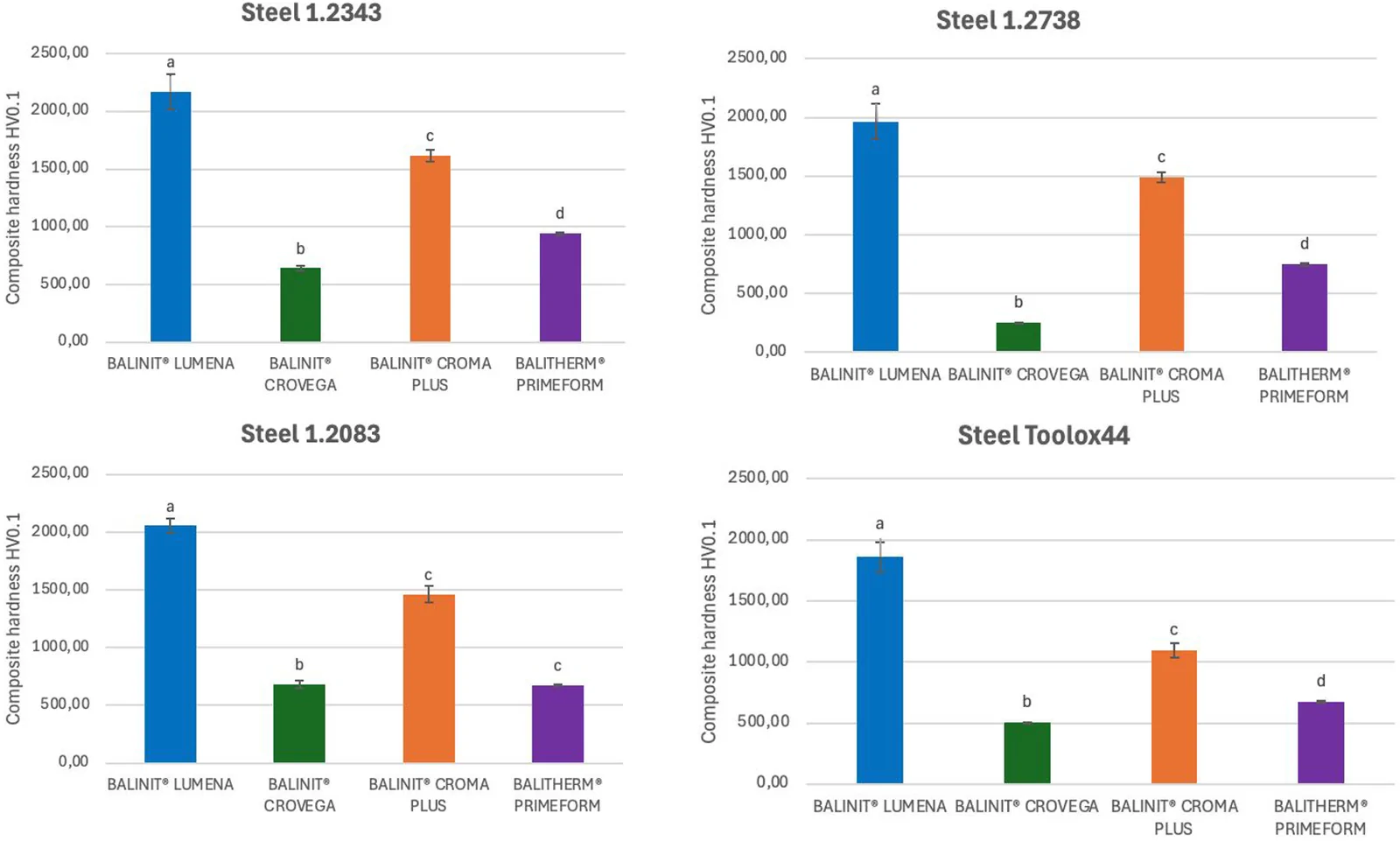
Vickers hardness (HV0.1) for different coatings deposited on selected steel grades: 1.2343, 1.2083, 1.2378 and Toolox 44. Values are presented as mean ± s.d. (*n* = 3). Different letters indicate statistically significant differences between coatings according to Tukey's HSD test (*α* = 0.05).

In addition to HV1 measurements, Vickers microhardness tests were also carried out under a lower load (HV0.1), allowing for a more surface-sensitive evaluation of the coating properties (figure 25). The comparison of HV0.1 and HV1 results provides important insight into the mechanical response of the coating–substrate system. For all investigated steel grades (1.2343, 1.2083, 1.2378 and Toolox 44), the HV0.1 values were significantly higher than the corresponding HV1 results. This difference is attributed to the well-known indentation size effect, as well as to the increasing contribution of the substrate at higher loads. At HV0.1, the indentation depth is limited, and the measured hardness primarily reflects the intrinsic properties of the coating. By contrast, at HV1, the indentation penetrates deeper into the material, resulting in a composite response influenced by both the coating and the substrate. Consequently, the measured hardness decreases and becomes more dependent on the mechanical properties of the underlying steel. Despite these differences, the relative ranking of coatings remained consistent across both loads, with BALITHERM® PRIMEFORM showing the highest hardness, followed by BALINIT® LUMENA, BALINIT® CROMA PLUS and BALINIT® CROVEGA. This confirms that the intrinsic strengthening mechanisms of the coatings dominate the overall mechanical response.

To evaluate the influence of coating type on hardness, a one-way ANOVA was performed separately for each steel grade and for both applied loads (HV0.1 and HV1; table 3 and table 4). The results revealed a highly significant effect of coating type on hardness (*p* < 0.001) for all investigated steels, confirming that the differences observed between coatings are not owing to random variation but are statistically meaningful.

The *F*-values obtained for individual steel grades were relatively high (e.g. *F* > 200 for HV0.1 and even higher for HV1 in some cases), indicating a strong between-group variance compared to within-group variability. This suggests that the type of coating is the dominant factor controlling hardness, regardless of the substrate material.

Post hoc analysis using Tukey's HSD test revealed statistically significant differences between most coating variants. Note, however, that the ranking of coatings depends on the applied load. For HV0.1 measurements, BALINIT® LUMENA (TiAlN) exhibited the highest hardness values, forming a distinct statistical group, which reflects its high intrinsic hardness. By contrast, for HV1 measurements, BALITHERM® PRIMEFORM consistently showed the highest hardness and formed a separate statistical group. This behaviour is attributed to the diffusion-based nature of the process, which enhances not only the surface but also the subsurface region, increasing load-bearing capacity and reducing substrate influence.

### 3.5. Corrosion test—hydrothermal incubation

Based on table 5 the corrosion resistance of all coatings incubated for 14 days in distilled water at 80°C was analysed. During the experiment, pH and electrical conductivity measurements of the solution were performed on days 1, 7 and 14, while the presence of corrosion was visually checked every day.

**Table 4.** One-way ANOVA results for microhardness (HV0.1).

steel grade	*F* (3,8)	*p*-value	significance
**1.2343**	218.73	5.15 × 10^−8^	***
**1.2083**	515.25	1.73 × 10^−9^	***
**1.2378**	280.50	1.93 × 10^−8^	***
**Toolox 44**	249.37	3.07 × 10^−8^	***

*** *p* < 0.001.

**Table 5.** The corrosion resistance of all coatings incubated for 14 days in distilled water at 80°C.

coating	type of steel	1 day	7 days	14 days
**CROVEGA**	1.2343			
	1.2083			
	1.2378			
	Toolox 44			
**LUMENA**	1.2343			
	1.2083			
	1.2378			
	Toolox 44			
	**CROMA PLUS**	1.2343			
		1.2083			
1.2378				
Toolox 44				
**PRIMEFORM**		1.2343			
		1.2083			
	1.2378			
	Toolox 44			

#### 3.5.1. Incubation for BALINIT CROVEGA coatings

In corrosion resistance tests of the BALINIT CROVEGA coating over a period of 14 days, clear signs of surface degradation were observed already in the early stages of the experiment. Measurements of pH and conductivity on day 1 showed a pH of 6.58–7.15 and conductivity of 12.4–74.8 µS cm^−1^. Visual observations showed the first signs of corrosion as early as day 3 of incubation. After 14 days of the experiment, all samples showed corrosion, the intensity of which corresponded to moderate corrosion. At that time, pH values ranged from 6.06 to 7.13, while conductivity reached values in the range 25 to 36 µS cm^−1^. Visual observation showed that the CROVEGA coating has low corrosion resistance under high-temperature water incubation conditions, with the rapid appearance of corrosion spots and their further progression over time.

#### 3.5.2. Incubation for BALINIT LUMENA coatings

In the case of the BALINIT LUMENA coating, the incubation process showed moderate corrosion resistance. On day 1 of the experiment, the pH values of the solution ranged from 6.18 to 6.36, and the conductivity was 11.5–27.4 µS cm^−1^. The first signs of visual corrosion were only observed on day 6, and the phenomenon was described as slight. This phenomenon was accompanied by a slight decrease in pH (to 5.79–6.48) and a slight increase in the conductivity of the solution within the range of 14.7–28.6 µS cm^−1^. On the tenth day of incubation, corrosion was already visible on all samples, with its intensity varying from slight to moderate. In the last measurement (day 14), the pH values ranged from 6.25 to 6.69, and the conductivity reached values of 23.5–50.8 µS cm^−1^. The results obtained indicate that the BALINIT LUMENA coating has moderate corrosion resistance. This coating therefore exhibits favourable protective properties under short-term exposure conditions, but its long-term stability is limited.

#### 3.5.3. Incubation for BALINIT CROMA PLUS coatings

In the case of the BALINIT CROMA PLUS coating, the incubation test showed the highest corrosion resistance of all the coatings analysed. On the first day, the pH values ranged from 5.23 to 5.78, and the conductivity reached 8.1–12.1 µS cm^−1^. On day 7, pH values remained between 5.58 and 5.89, and conductivity ranged from 10.6 to 18.5 µS cm^−1^, confirming the electrochemical stability of the solution, indicating a low ion content in the solution and no intense degradation processes. After 14 days of the experiment, the pH of the samples did not change. During the entire incubation period, no signs of visual corrosion were found on the surface of the samples. Changes in pH and conductivity should therefore be attributed to natural ion exchange processes between the material and the test environment, rather than to intensive corrosion. The absence of visual surface degradation, stable electrochemical parameters and no loss of mass demonstrates the high corrosion resistance of the BALINIT CROMA PLUS coating. The results clearly indicate that the CrN layer is the most durable protective system under the incubation conditions tested, providing an effective barrier against the effects of the aqueous environment at elevated temperatures.

#### 3.5.4. Incubation for BALITHERM PRIMEFORM coatings

In the case of the BALITHERM PRIMEFORM coating, the incubation test showed clearly limited corrosion resistance. Already on the first day of the experiment, the solution parameters indicated chemical destabilization—pH values dropped to 4.45–5.17 and conductivity ranged from 7.9 to 15.7 µS cm^−1^, suggesting ion exchange at the phase boundary. Visual inspection confirmed the appearance of the first signs of extensive corrosion after just 1 day of incubation, and on the third day, strong and intense degradation processes were observed, classified as very intense corrosion. On day 7, pH values ranged from 5.66 to 6.52, and conductivity increased to a maximum of 39.7 µS cm^−1^. Subsequent stages of the experiment confirmed further corrosion progression—on the tenth day, all samples showed clear signs of degradation, and on day 14, the pH values (6.26–6.87) and conductivity values (18.8–48.7 µS cm^−1^) obtained confirmed the continuing high corrosion activity. The results obtained indicate that the BALITHERM PRIMEFORM coating is characterized by very low corrosion resistance. The early onset of corrosion processes, rapid intensification of degradation and changes in solution parameters distinguish it from other coatings and clearly disqualify it as an effective protective barrier in long-term exposure to hydrothermal conditions.

### 3.6. Tribology test

Tribological tests conducted in reciprocating motion have shown that the type of base steel significantly affects the behaviour of BALINIT LUMENA and BALINIT CROMA PLUS coatings (figures 26–42).

In the case of the BALINIT LUMENA coating, the most favourable results were obtained for steel 1.2378 HH, where the friction coefficient was the lowest and most stable during the test. This means that the coating effectively reduced adhesive wear mechanisms, ensuring an even distribution of load across the contact surface. Steel 1.2343 also showed stable COF behaviour, but the values were slightly higher than for steel 1.2378. This indicates that the coating interacts correctly with the substrate but is less effective in reducing friction. For Toolox 44, average COF values were recorded, stable throughout the test. The topography of the wear surface indicates uniform abrasion, confirming that this steel-coating combination provides moderate tribological resistance. The least favourable results were obtained for steel 1.2083, where the COF reached higher values, and its course was less stable. The surface after the test showed local deformations and more intense wear.

Similar relationships were observed for the BALINIT CROMA PLUS coating. The best tribological results were obtained for 1.2378 HH, where the COF was lowest and the wear pattern most uniform. The wear surface was characterized by a shallow depth and even distribution, which indicates very good compatibility between the coating and the substrate.

Steel 1.2343 again showed good properties—the COF curve was stable, but the values were slightly higher than for 1.2378. In the case of Toolox 44, average COF values were recorded, with the wear topography indicating moderate intensity of the process.

The least favourable results, as in the LUMENA series, were obtained for steel 1.2083. The COF took on the highest values, and the course was characterized by greater fluctuations, which were associated with the presence of local surface damage and lower stability in tribological contact.

The best tribological properties are exhibited by the combination of BALINIT (LUMENA and CROMA PLUS) with 1.2378 HH steel. 1.2343 and Toolox 44 steels deliver average results—stable COF curves, but without values as favourable as those of 1.2378. Steel 1.2083 is the least suitable substrate for the coatings tested—it exhibits the highest friction and the lowest wear resistance. The type of base steel is a critical factor determining the tribological properties of PVD coatings, and the choice of coating should always be related to the microstructure and mechanical properties of the substrate material.

**Figure 26.**
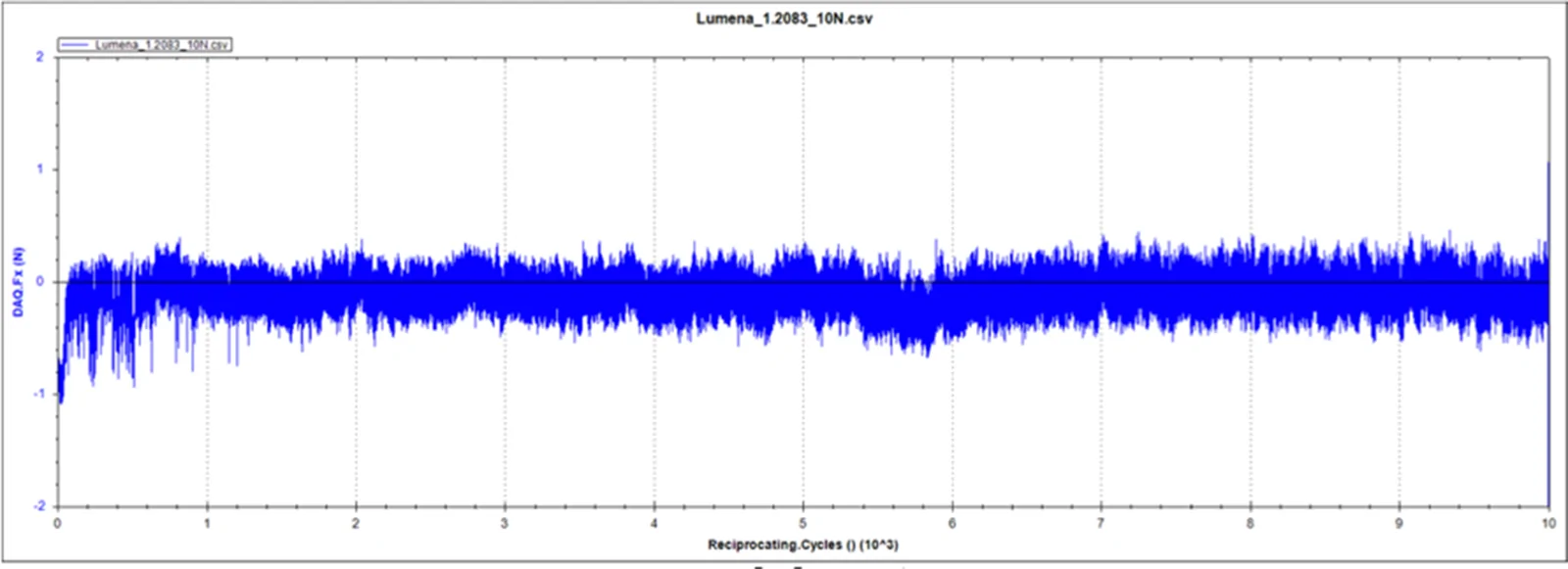
Change in friction force *F_x_* (N)—LUMENA cover on sample 1.2083 50 HRC.

**Figure 27.**
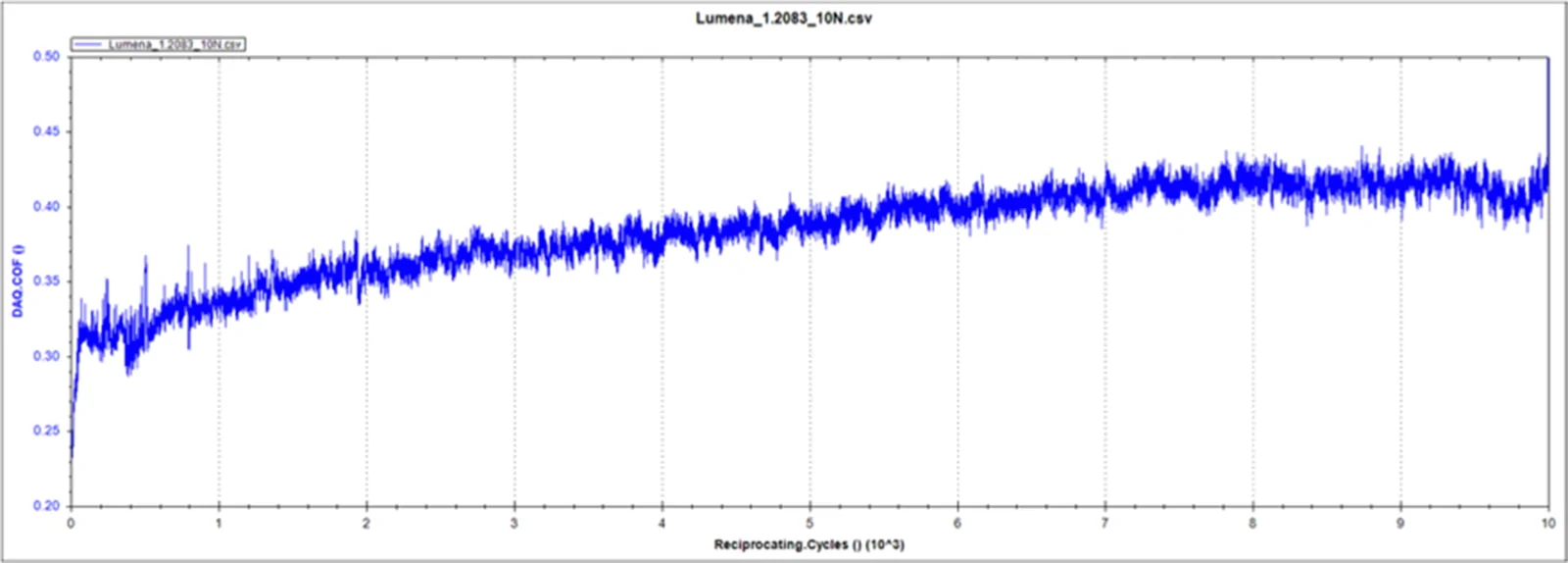
Change in the coefficient of friction COF—LUMENA cover on sample 1.2083 50 HRC test.

**Figure 28.**
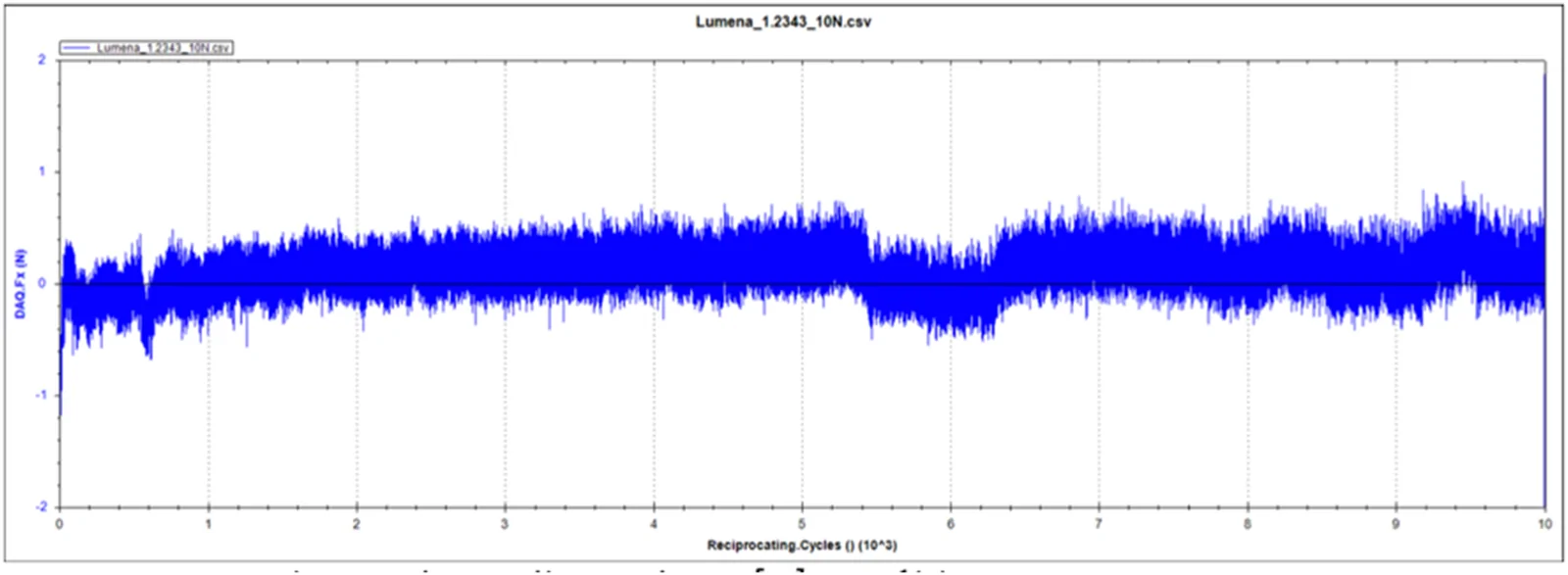
Change in friction force *F_x_* (N)—LUMENA cover on sample 1.2343 50 HRC.

**Figure 29.**
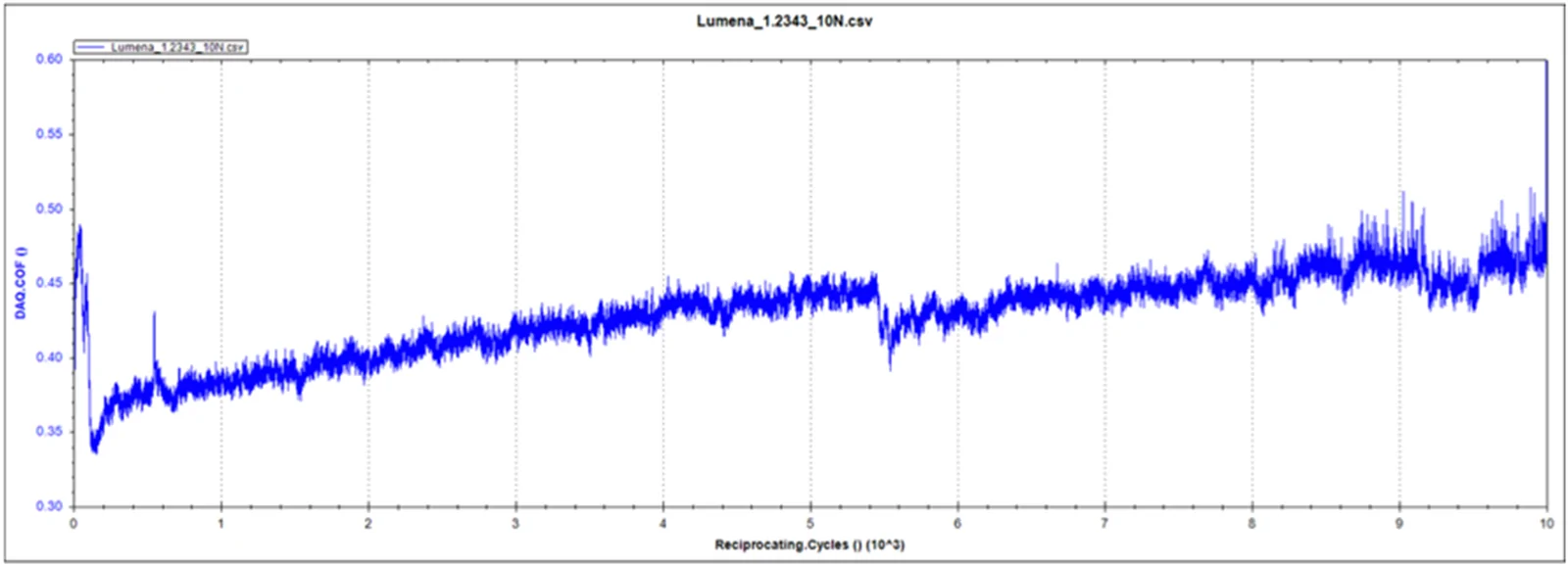
Change in the COF—LUMENA cover on sample 1.2343 50 HRC test.

**Figure 30.**
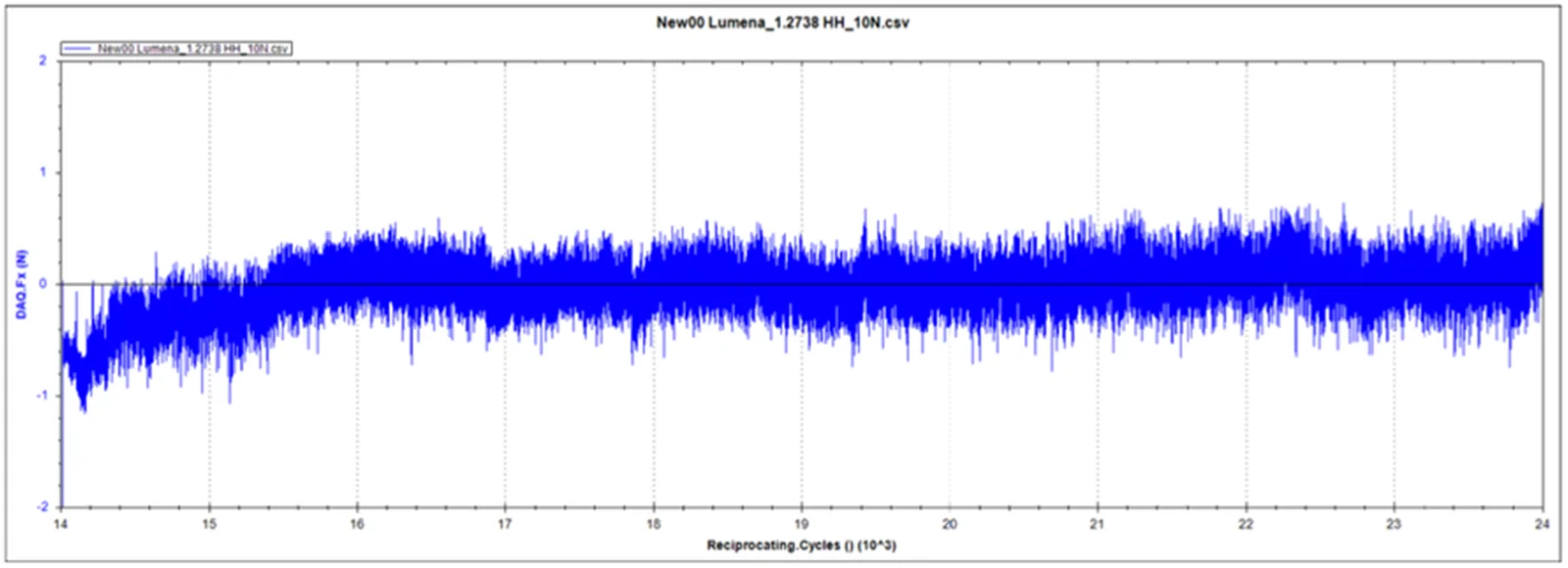
Change in friction force *F_x_* (N)—LUMENA cover on sample 1.2378 HH.

**Figure 31.**
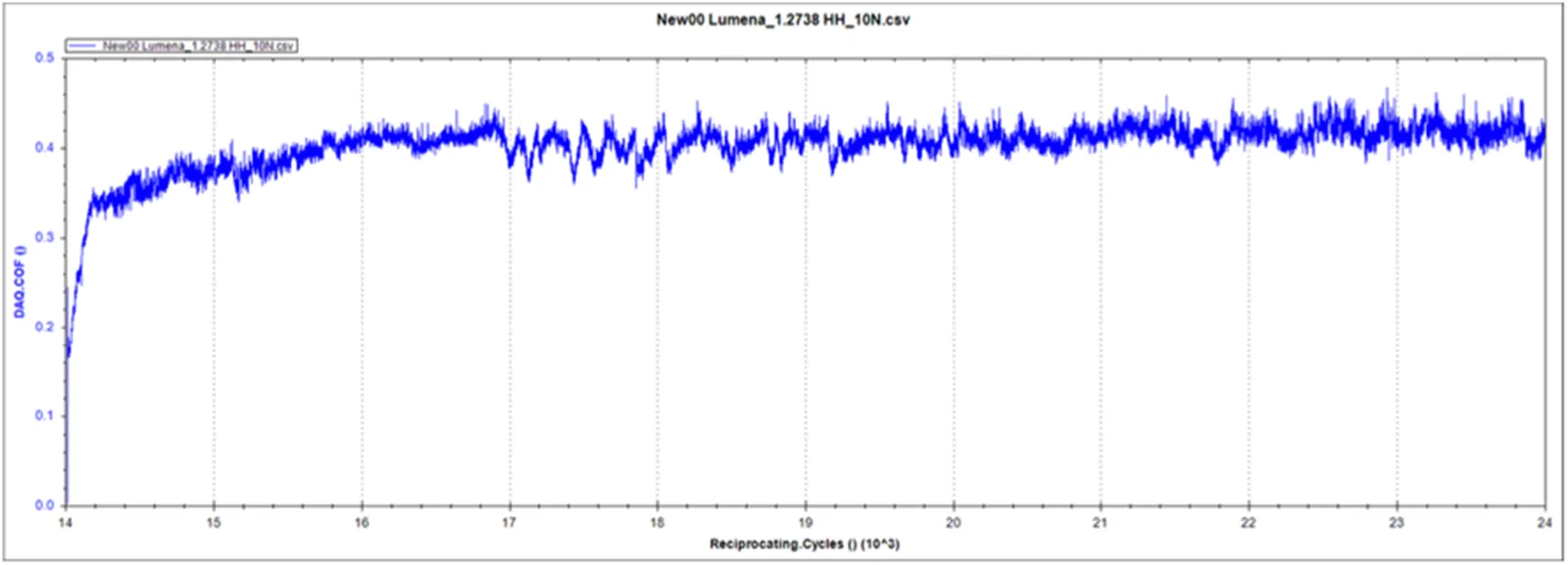
Change in the coefficient of friction COF—LUMENA cover on sample 1.2378 HH test.

**Figure 32.**
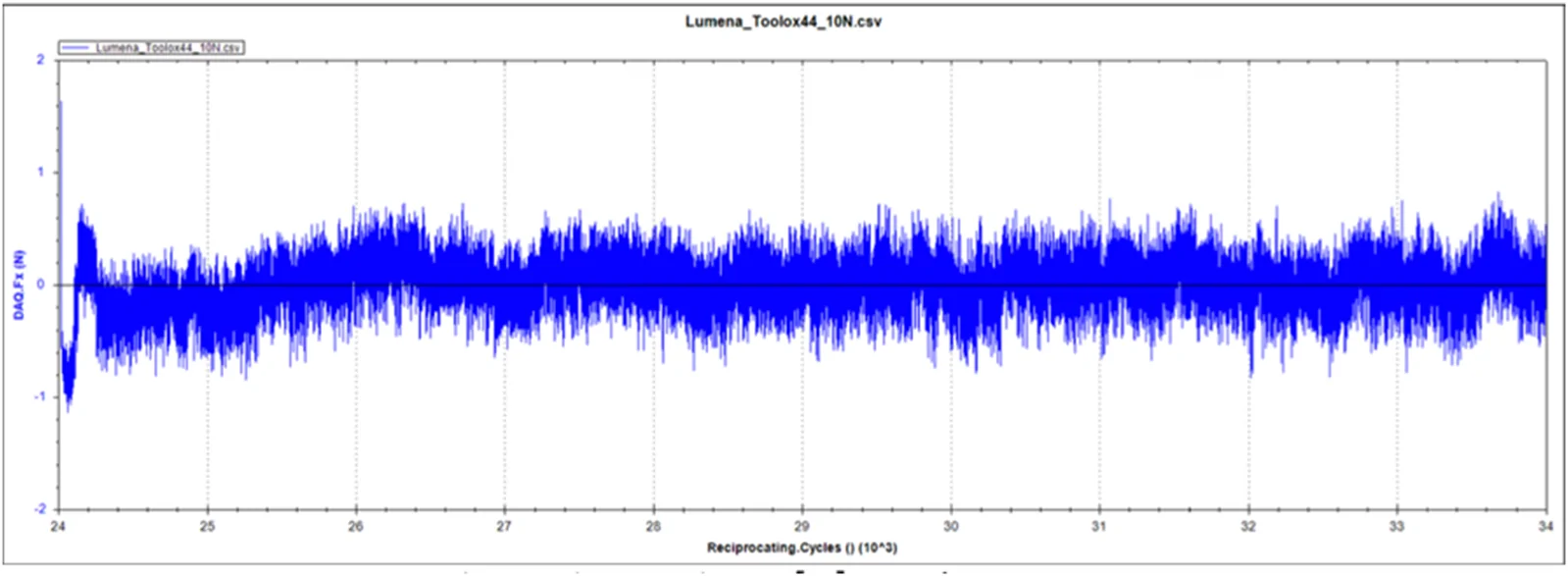
Change in friction force *F_x_* (N)—LUMENA cover on sample Toolox 44.

**Figure 33.**
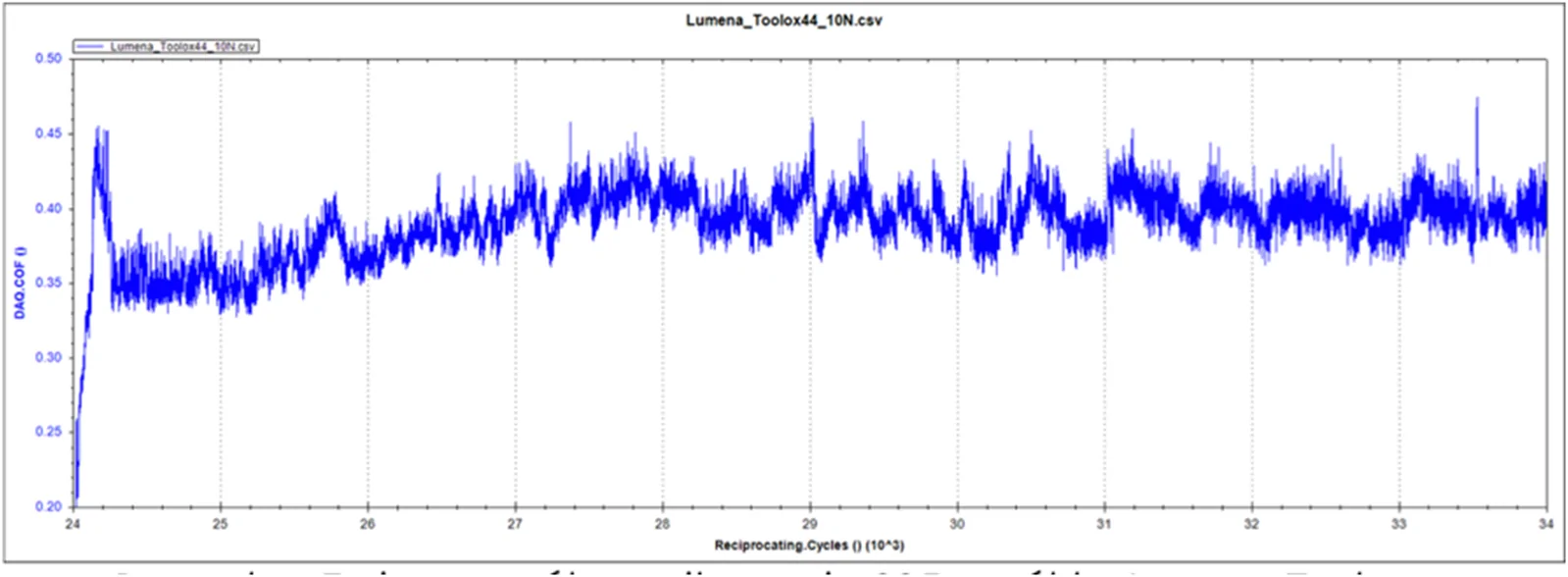
Change in the COF—LUMENA cover on sample Toolox 44.

**Figure 34.**
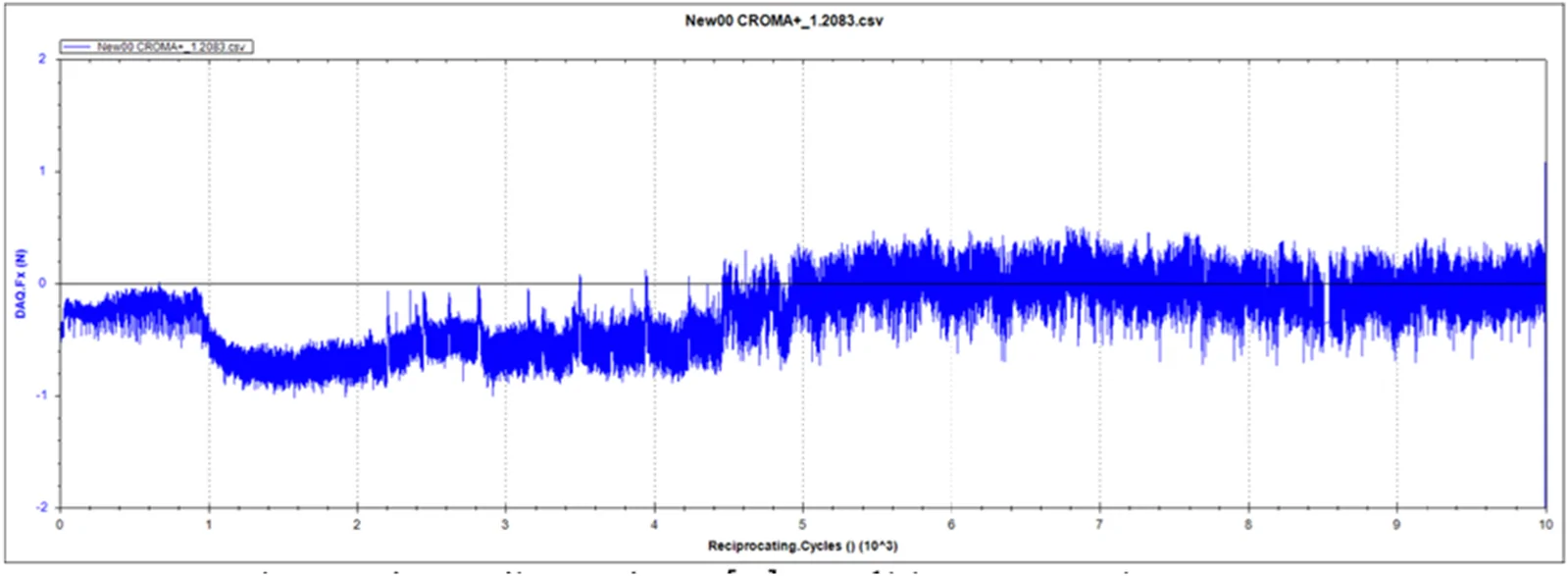
Change in friction force *F_x_* (N)—CROMA PLUS cover on sample 1.2083 50 HRC.

**Figure 35.**
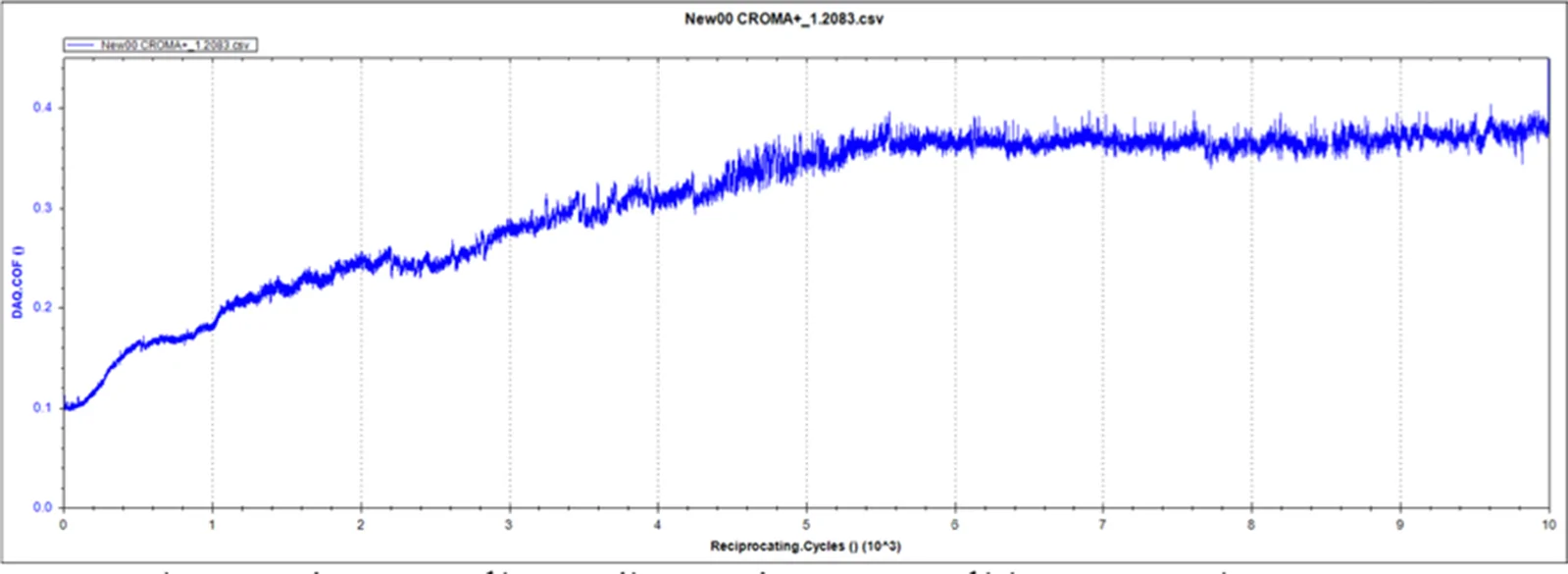
Change in the coefficient of friction COF—LUMENA cover on sample 1.2083 50 HRC.

**Figure 36.**
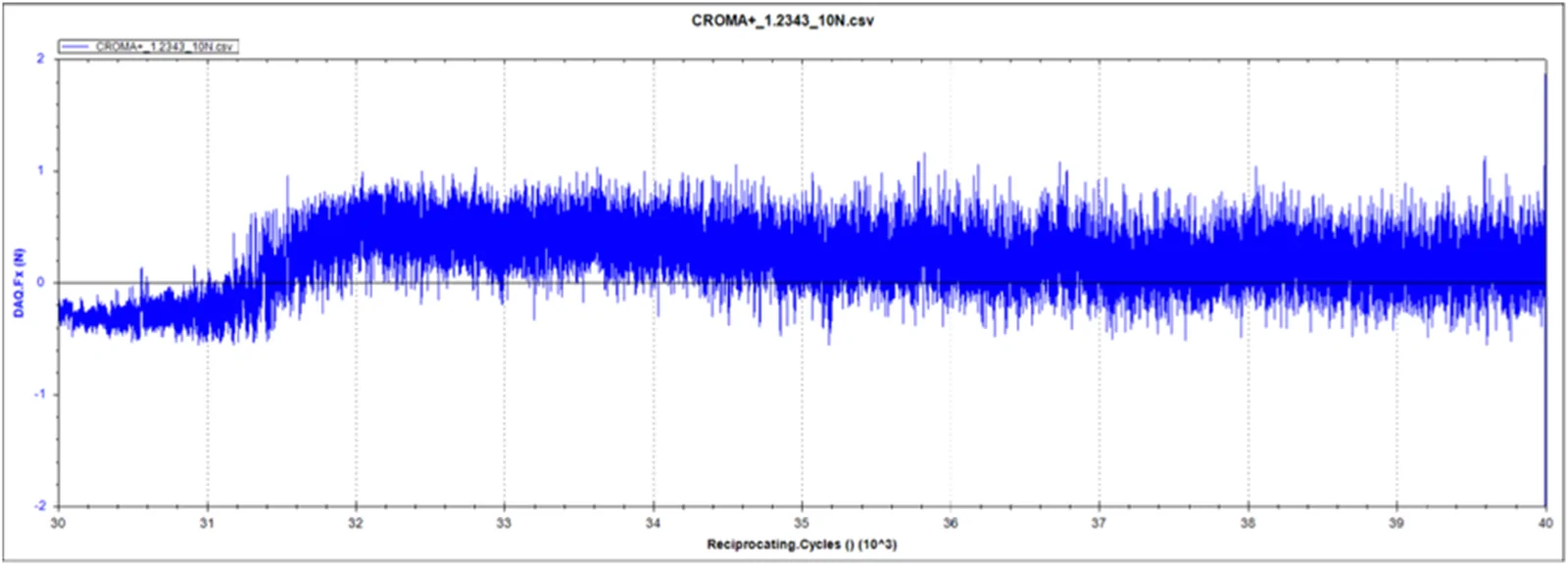
Change in friction force *F_x_* (N)—CROMA PLUS cover on sample 1.2343 50 HRC.

**Figure 37.**
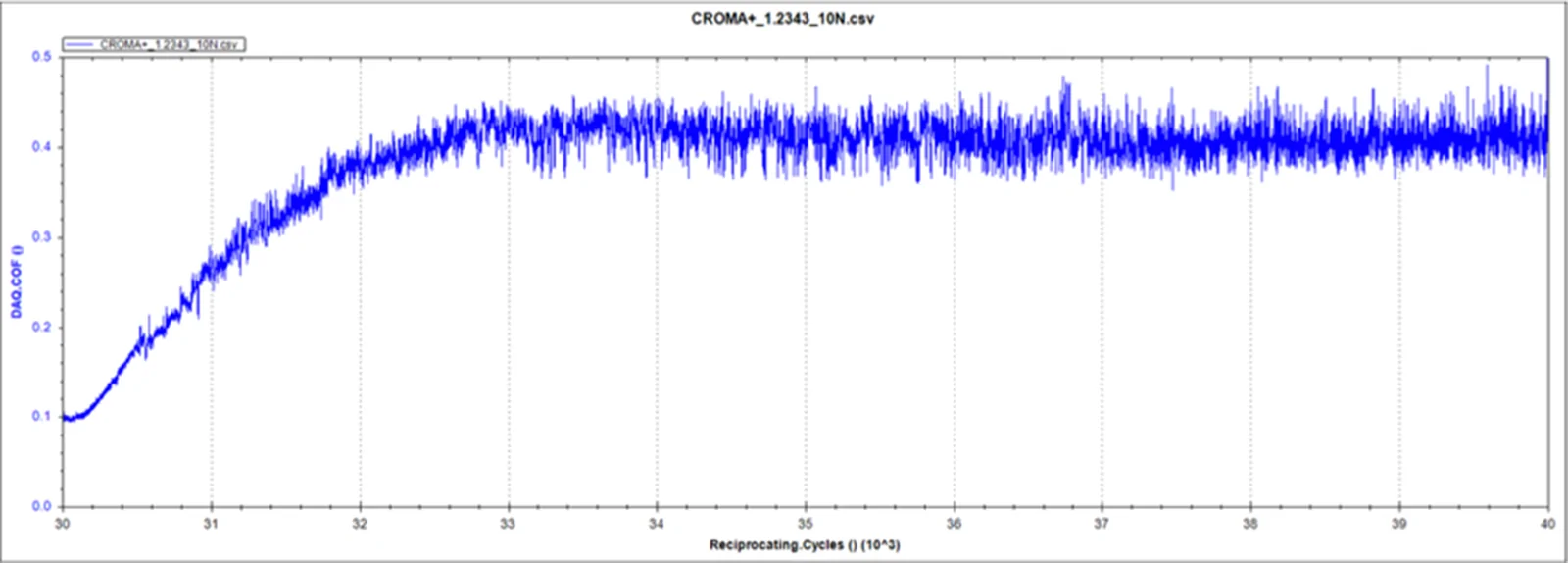
Change in the COF—CROMA PLUS cover on sample 1.2343 50 HRC.

**Figure 38.**
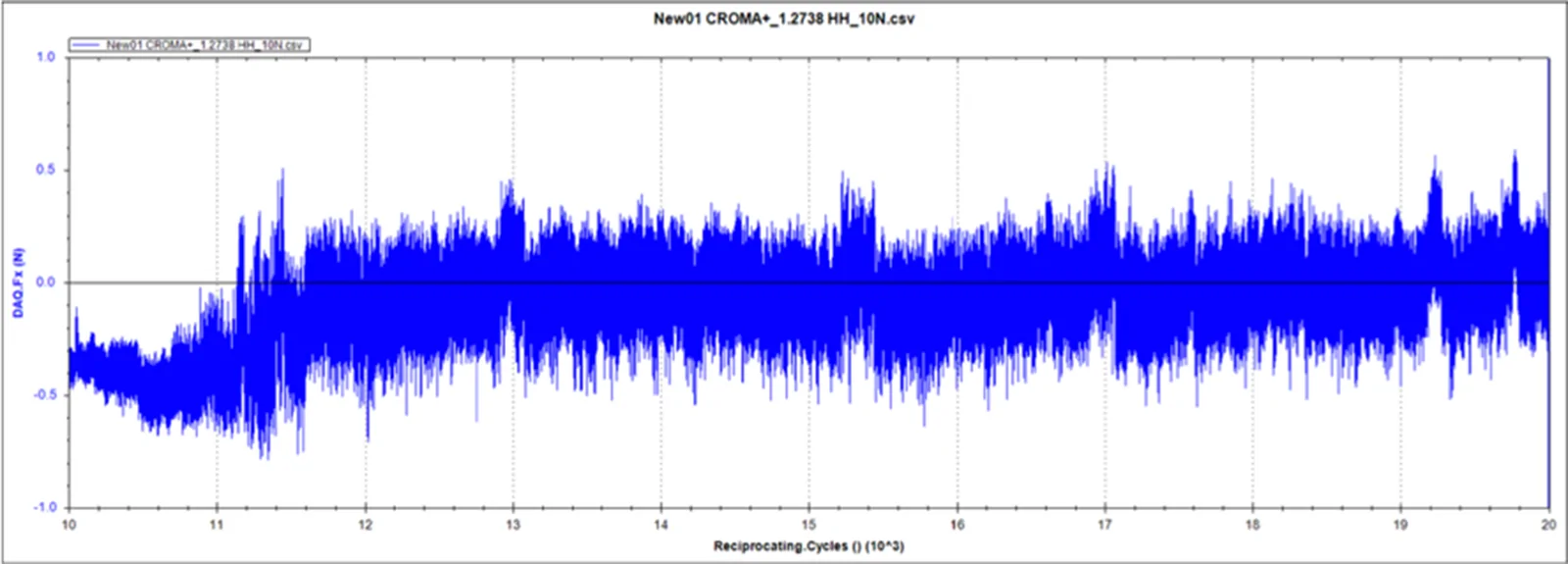
Change in friction force *F_x_* (N)—CROMA PLUS cover on sample 1.2378 HH.

**Figure 39.**
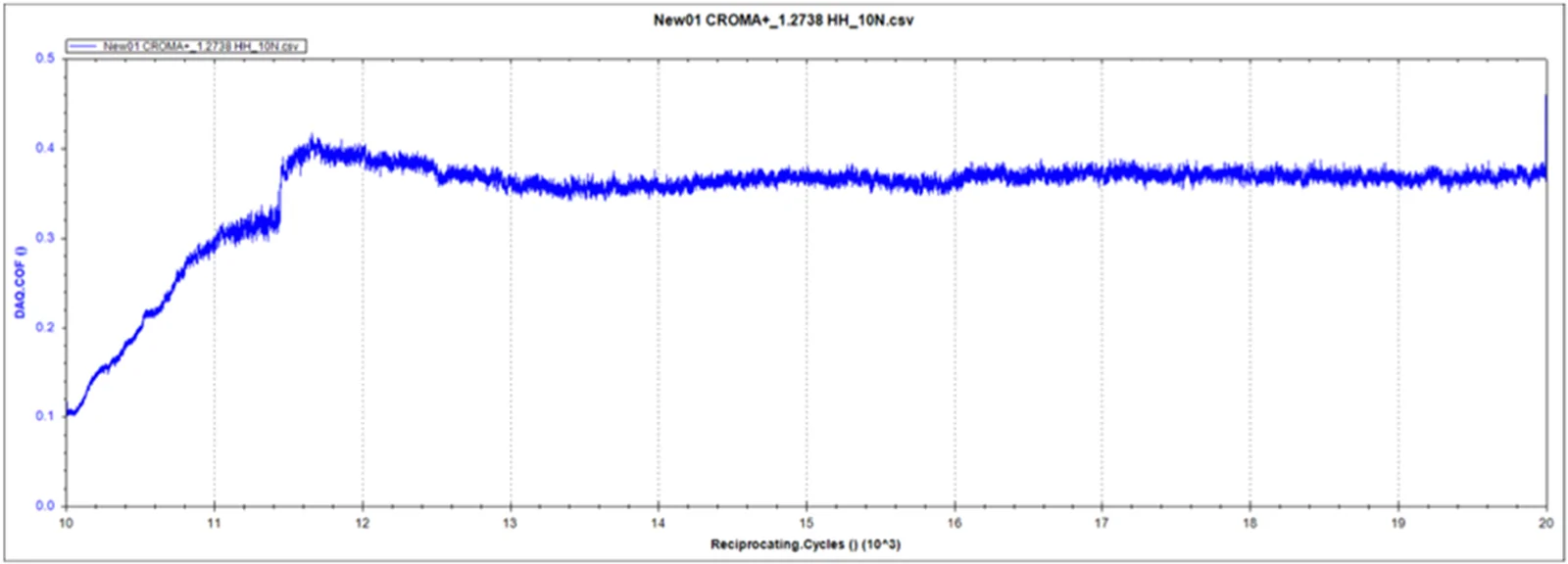
Change in the coefficient of friction COF—CROMA PLUS cover on sample 1.2378 HH.

**Figure 40.**
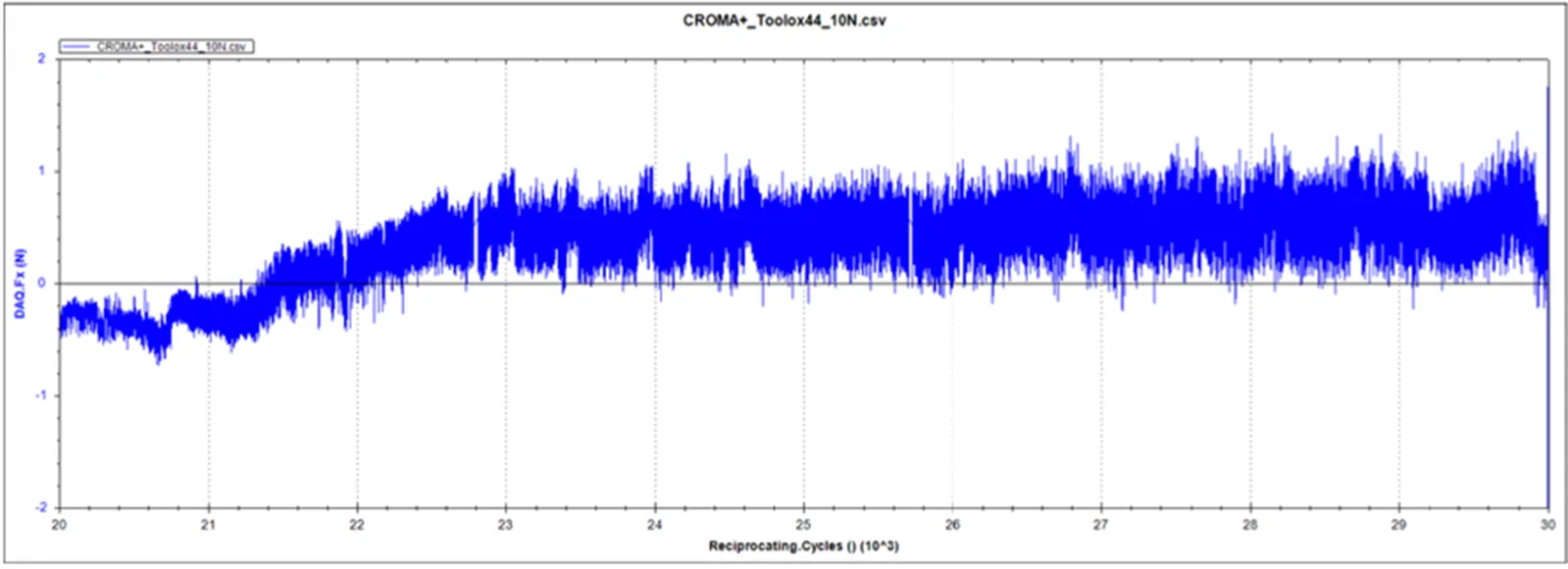
Change in friction force *F_x_* (N)—CROMA PLUS cover on sample Toolox 44.

**Figure 41.**
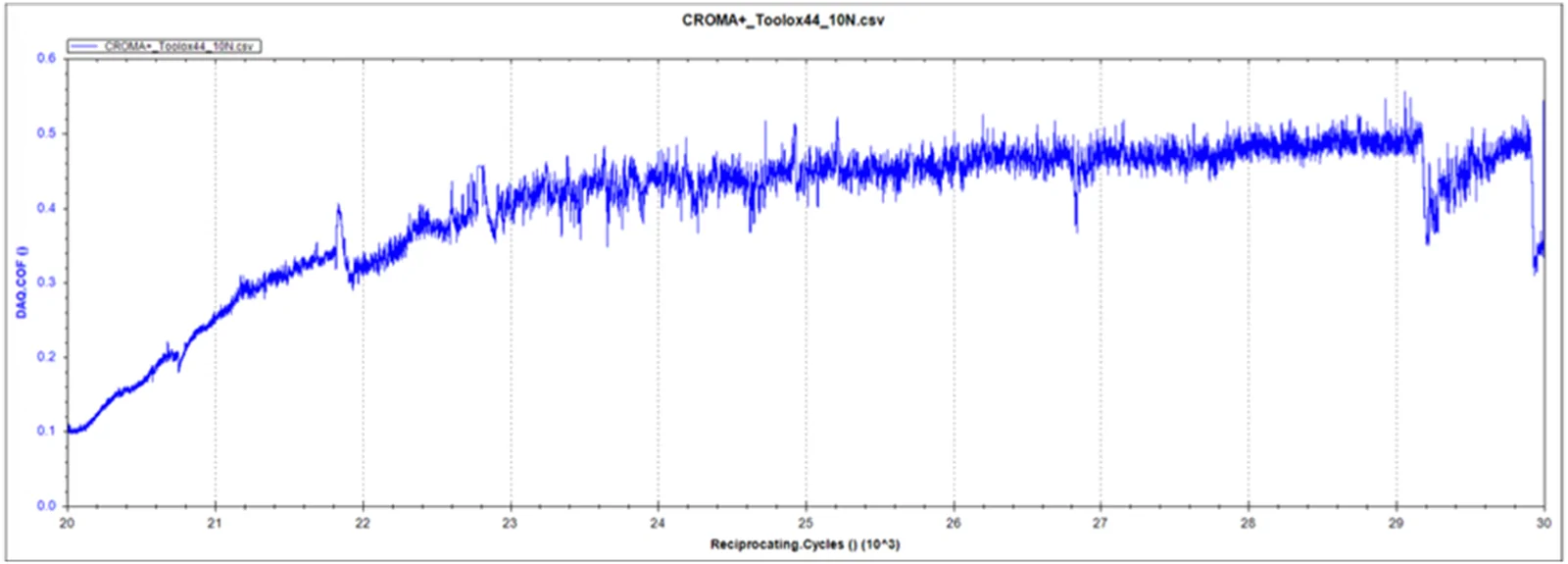
Change in the COF—CROMA PLUS cover on sample Toolox 44.

**Figure 42.**
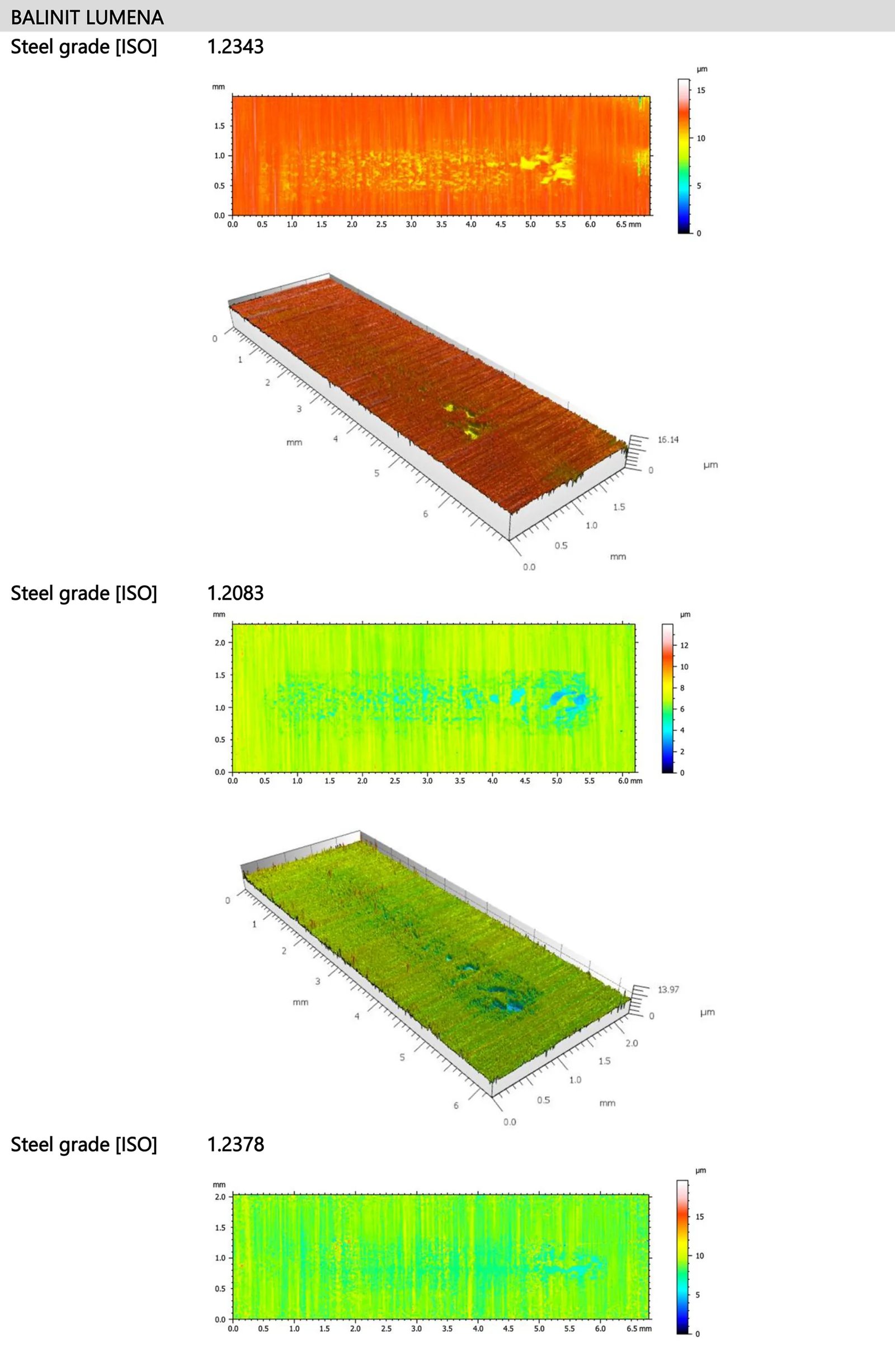

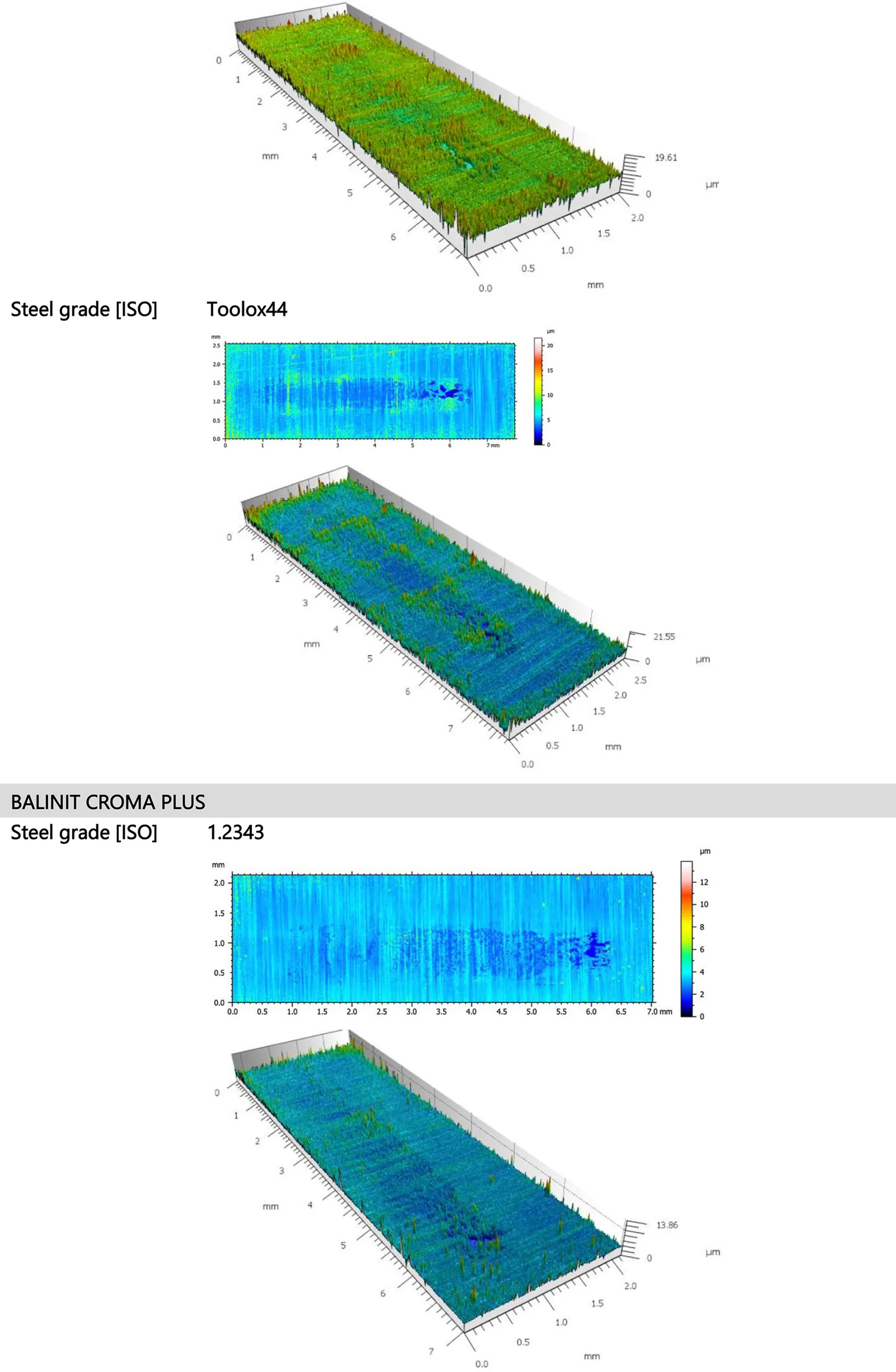

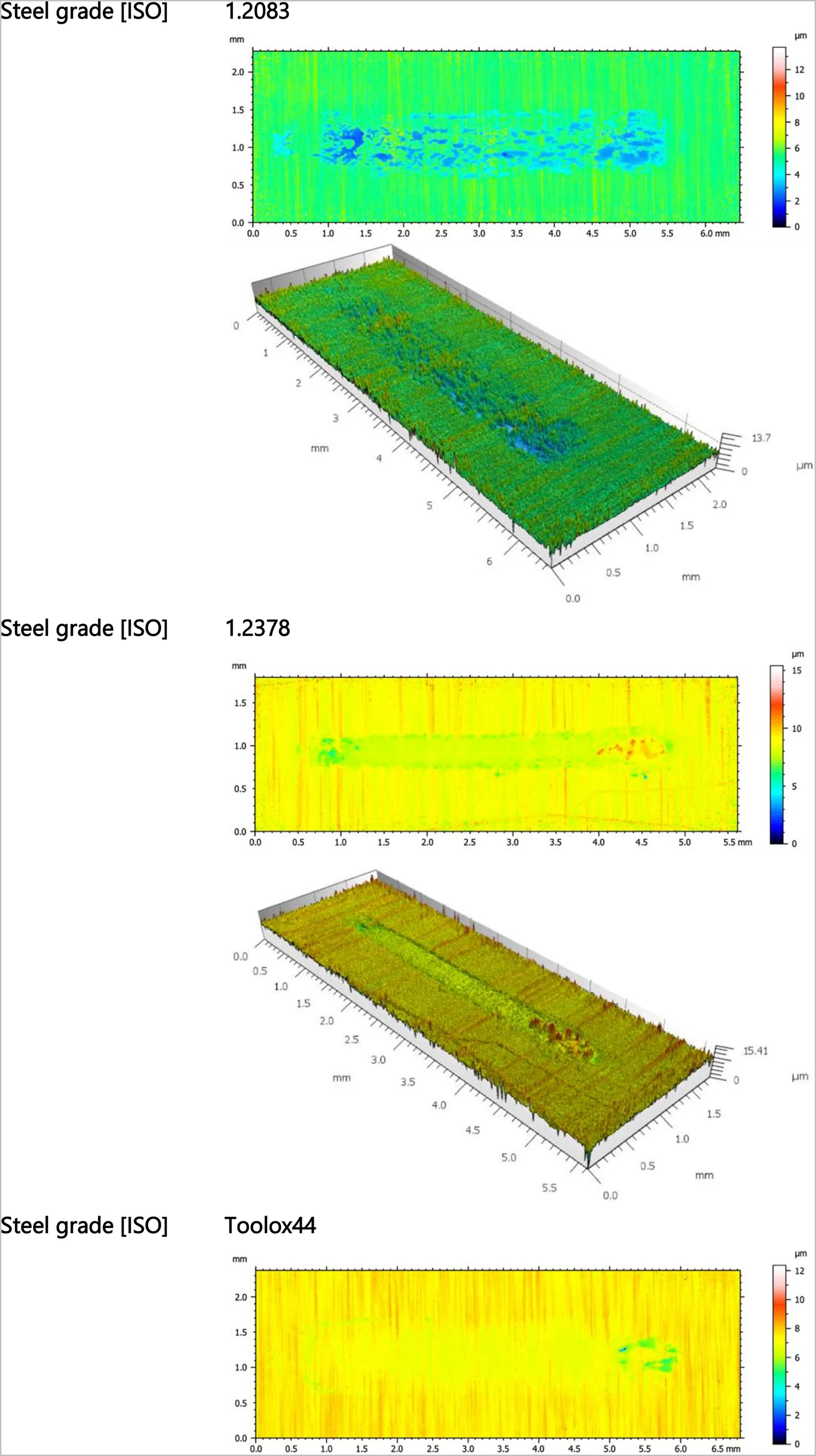

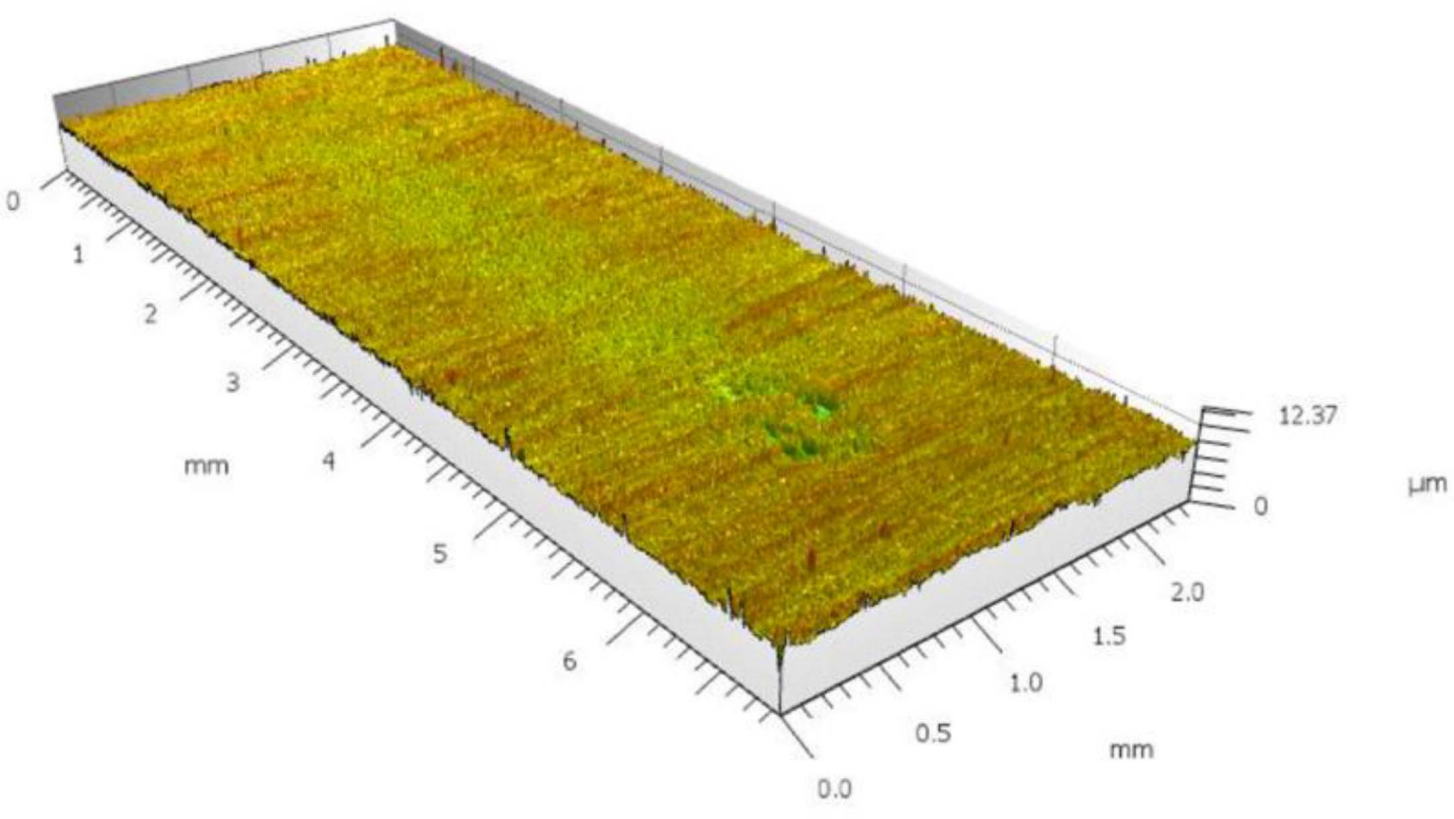
Topography of the wear surface of the tested samples.

### 3.7. X-ray fluorescence analysis

Analysis of the chemical composition of tool steel 1.2378 using XRF for substrates with various coatings showed clear differences in the proportion of the main alloying elements (table 6). In the case of the CROVEGA coating, the dominant component was iron (Fe—72.97%) with a significant proportion of chromium (Cr—24.59%), which is characteristic of high chromium steels. In turn, steel with a CROMA PLUS coating was characterized by an even higher chromium content (Cr—33.06%) with a reduced iron content (Fe—64.44%), which indicates potentially greater passivation potential and better corrosion resistance. The composition of steel coated with BALITHERM PRIMEFORM was different, with iron (Fe—91.75%) dominating and a low chromium content (Cr—5.04%); this configuration suggests lower corrosion resistance of the material, despite the presence of molybdenum (Mo—1.35%) and other alloying additives. The most diverse composition was observed in the case of the LUMENA coating, where titanium (Ti—42.76%) was the main element, while the content of aluminium (Al—20.58%) and iron (Fe—34.93%) indicated significantly different chemical characteristics compared to other coating systems.

The results clearly indicate that, depending on the coating used, 1.2378 steel is characterized by a different proportion of alloying elements important for corrosion resistance. The most favourable properties are potentially provided by the high chromium content in the CROMA PLUS system, while the presence of titanium and aluminium in the LUMENA coating suggests additional reinforcement of resistance to oxidation at high temperatures. On the other hand, the low chromium content in the PRIMEFORM system correlates with the lower corrosion resistance observed in incubation tests.

**Table 6.** Analysis of the chemical composition of tool steel 1.2378.

Steel 1.2378							
BALINIT crovega		BALINIT Croma Plus		Balitherm primeform		BALINIT Lumena	
formula	concentration %	formula	concentration %	formula	concentration %	formula	concentration %
Fe	72.97	Fe	64.44	Fe	91.75	Ti	**42.76**
Cr	**24.59**	Cr	**33.06**	Cr	**5.04**	Fe	34.93
Mo	1.19	Mo	1.15	Mo	1.35	Al	**20.58**
Al	0.43	Al	0.53	Si	0.51	Cr	0.63
Mn	0.23	V	0.22	Al	0.45	Mn	0.43
V	0.23	Mn	0.21	Mn	0.32	Ni	0.28
Ni	0.12	Ca	0.15	V	0.26	Mo	0.14
Cu	0.06	Ni	0.11	Ni	0.17	Ca	0.06
W	0.06	Br	0.05	Cu	0.08	Cu	0.05
Ca	0.05	W	0.04	W	0.03	W	0.03
Br	0.04	Bi	0.02	Br	0.02	Sc	0.03
Bi	0.02	Pb	0.01	Bi	0.02	Bi	0.03
Pb	0.01					Au	0.02
						Pt	0.01
						Pb	0.01
						Hg	0.01

### 3.8. Variable angle spectroscopic ellipsometry spectroscopic analysis

**Figure 43.**
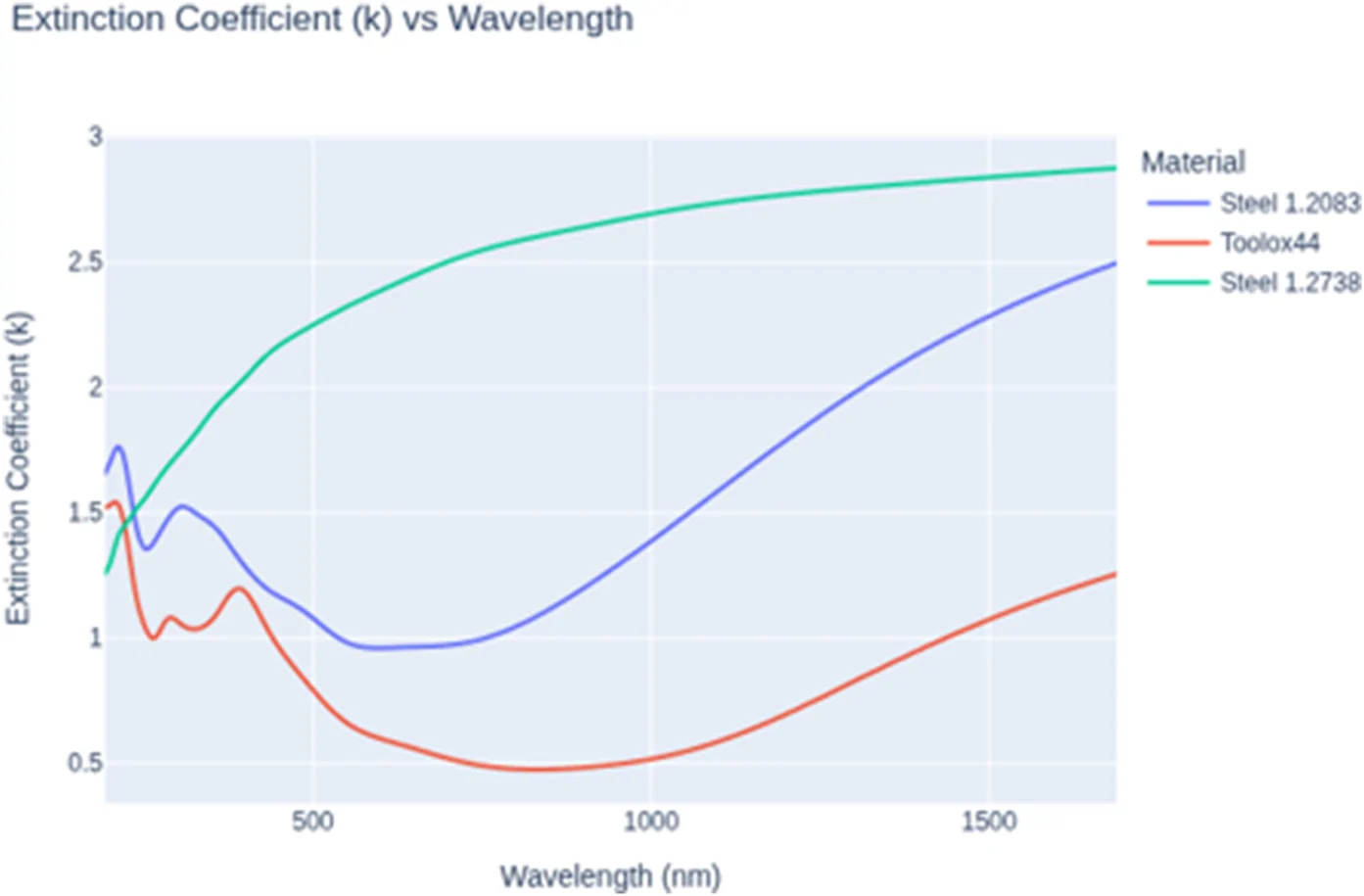
Graph of the dependence of the extinction coefficient (*k*) on wavelength.

Optical characterization of thin-film coatings deposited on three steel substrates (1.2083, Toolox 44 and 1.2378) was performed using VASE in the wavelength range of 192–1688 nm ( figure 43). The refractive index (*n*) and extinction coefficient (*k*) were extracted from model fitting and analysed across ultraviolet (UV), visible (VIS) and near-infrared (NIR) spectral regions.

Steel grade 1.2083:

— UV: *n* = 1.96–3.04, *k* = 1.29–1.76,
— VIS: *n* = 3.04–3.15, *k* = 0.96–1.28,
– NIR: *n* = 2.99–4.18, *k* = 0.97–2.50.

Steel grade Toolox 44:

— UV: *n* = 1.88–2.69, *k* = 1.00–1.54,
— VIS: *n* = 2.70–2.98, *k* = 0.52–1.18,
— NIR: *n* = 2.91–3.40, *k* = 0.47–1.26.

Steel grade 1.2378:

— UV: *n* = 1.18–1.96, *k* = 1.26–2.04,
— VIS: *n* = 1.96–2.96, *k* = 2.04–2.50,
— NIR: *n* = 2.97–5.24, *k* = 2.51–2.88.

All samples exhibited strong dispersion in the UV region, with decreasing *k* values towards the VIS and NIR ranges, indicating reduced absorption and increased transparency. The refractive index stabilized in the NIR region, suggesting consistent optical behaviour at longer wavelengths. These results confirm the suitability of the coatings for applications requiring optical stability and low absorption in the visible and infrared domains.

### 3.9. Gloss analysis

The tests were conducted on four samples, one for each of the selected coatings. Each measurement was repeated three times at different locations on the test samples to maximize the reliability of the results. The results listed in table 7 show the average findings for both measuring devices used in the tests.

**Table 7.** Gloss for the tested coatings.

**coating**	**3Color GM-38**		**ZGM 1120**
	angle 20°	angle 60°	angle 20°
CROVEGA	16.8	64.6	13.3
CROMA PLUS	3.0	25.0	8.2
LUMENA	8.6	39.4	9.9
PRIMEFORM	-	-	38.4

Gloss measurements revealed noticeable differences between the investigated coating systems, depending on both the coating type and the measurement configuration. The results obtained using the 3Color GM-38 glossmeter (20° and 60° geometries) and the ZGM 1120 device (20° geometry) are listed in table 7. For measurements performed with the GM-38 device, BALINIT® CROVEGA exhibited the highest gloss values (64.6 at 60° and 16.8 at 20°), indicating a relatively smooth and optically reflective surface. BALINIT® LUMENA showed intermediate gloss values (39.4 at 60° and 8.6 at 20°), while BALINIT® CROMA PLUS demonstrated the lowest gloss (25.0 at 60° and 3.0 at 20°), suggesting increased light scattering owing to surface or structural features. Measurements performed using the ZGM 1120 device (20° geometry) confirmed a similar trend for PVD coatings, with CROVEGA (13.3) and LUMENA (9.9) exhibiting higher gloss than CROMA PLUS (8.2). Notably, the BALITHERM® PRIMEFORM coating exhibited the highest gloss value (38.4), indicating strong specular reflection characteristics. Note that not all coatings were measured under identical configurations owing to instrumental and surface-related limitations. Therefore, the results are primarily interpreted in terms of trends within each measurement system rather than direct quantitative comparison between devices.

## 4. Discussion

The experimental results demonstrate a strong interdependence between the type of coating, the substrate microstructure and the resulting mechanical and corrosion properties. SEM and optical analyses confirmed that surface morphology after coating deposition varies significantly depending on both steel grade and coating type. Among the PVD systems, BALINIT LUMENA (TiAlN) and BALINIT CROVEGA (CrN/TiN) formed uniform and adherent layers with limited microporosity, while BALINIT CROMA PLUS (CrN) exhibited a slightly higher number of microdefects and inclusions. Nevertheless, the CrN-based coating showed the best corrosion stability, suggesting that the presence of a continuous chromium-rich phase compensates for minor structural irregularities by promoting surface passivation. The microhardness results reveal that coating effectiveness depends mainly on the deposition mechanism. The diffusion-based BALITHERM PRIMEFORM treatment resulted in the highest hardness values (735–1119 HV1), owing to nitrogen enrichment of the surface layer and the formation of hard nitrides. However, its poor corrosion resistance indicates that the same diffusion pathways facilitating nitrogen penetration also promote electrochemical activity in aqueous media. By contrast, BALINIT LUMENA increased surface hardness by 30–70% depending on the substrate while maintaining moderate corrosion resistance and low roughness, confirming its suitability for applications requiring a balance between mechanical durability and dimensional precision. The tribological tests confirmed that steel 1.2378 HH is the most compatible substrate for PVD coatings. Both LUMENA and CROMA PLUS coatings on this steel exhibited the lowest and most stable COF, consistent with their smooth surface topography (Ra = 0.09–0.14 µm). A clear correlation between roughness and tribological behaviour was observed: lower Ra values were associated with lower and more stable COF curves, indicating reduced adhesive wear. Conversely, steels with higher roughness (1.2083, Toolox 44) exhibited greater friction fluctuations and localized damage, probably owing to micro-asperity interactions. Gloss measurements further indicate that optical performance is not governed solely by surface roughness but also by the intrinsic optical properties of the coating. In particular, the high gloss observed for the BALITHERM PRIMEFORM coating despite increased roughness suggests a strong contribution of its reflective surface characteristics. The corrosion incubation tests demonstrated a distinct hierarchy of coating stability. BALINIT CROMA PLUS remained completely free of corrosion after 14 days at 80°C, with minimal pH and conductivity changes, confirming the protective efficiency of CrN films. BALINIT LUMENA showed delayed degradation (visible after day 6), suggesting limited passivation and slower ion exchange kinetics. By contrast, BALINIT CROVEGA and especially BALITHERM PRIMEFORM suffered rapid degradation and solution acidification, which is consistent with their lower Cr content observed in XRF results. The combination of high chromium content (33 wt%) and uniform deposition makes CROMA PLUS the most chemically stable system among those tested.

Overall, the synergy between the coating type and substrate is crucial. TiAlN- and CrN-based coatings ensure a favourable balance between hardness, smoothness and corrosion resistance, while diffusion treatments offer superior mechanical reinforcement but at the cost of chemical stability. These observations are consistent with trends reported in recent literature [1,3,14], which emphasize the importance of microstructural compatibility and coating density in determining long-term tool performance.

**Figure 44.**
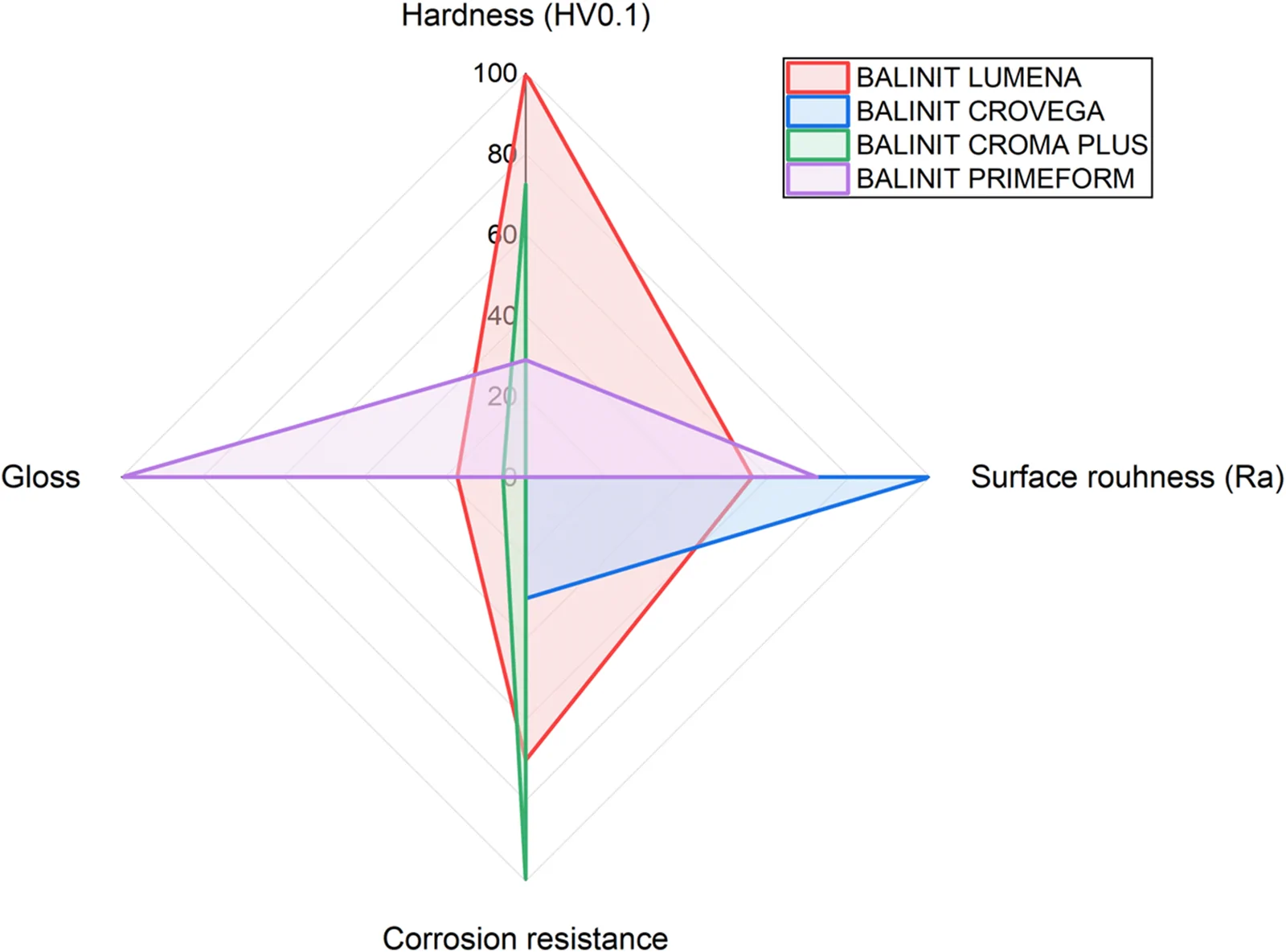
Multi-criteria performance comparison of the investigated coatings deposited on steel 1.2378 based on normalized microhardness (HV0.1), surface roughness (Ra), corrosion resistance and gloss values. All properties were normalized to a 0–100 scale, where higher values indicate better performance.

To facilitate a multi-criteria comparison of the investigated coating systems, the measured properties were normalized to a common 0–100 scale and presented as a radar chart (figure 44). The comparison was performed for steel grade 1.2378 to enable direct assessment of all coatings on the same substrate. The analysis demonstrates that no single coating simultaneously maximizes all investigated properties. BALINIT LUMENA exhibited the most balanced overall performance owing to its high microhardness, good corrosion resistance and satisfactory surface quality. BALINIT CROMA PLUS provided the highest corrosion resistance, BALINIT CROVEGA achieved the lowest surface roughness, whereas BALITHERM PRIMEFORM was distinguished by the highest gloss. Therefore, the selection of the most suitable coating depends on the specific functional requirements of the intended application.

## 5. Conclusion

The present study demonstrates that the performance of protective coatings applied to tool steels is governed by a complex interplay between deposition method, coating composition and substrate microstructure. The results clearly indicate that no single coating optimizes all functional properties simultaneously; instead, performance is determined by trade-offs between mechanical strength, corrosion resistance and surface topography. The diffusion-based BALITHERM® PRIMEFORM treatment produced the highest hardness values (up to 1119 HV1), reflecting its strong load-bearing capacity resulting from subsurface modification. However, its poor corrosion resistance under hydrothermal conditions limits its applicability in humid or chemically aggressive environments. By contrast, the TiAlN-based BALINIT® LUMENA coating provided a balanced combination of increased hardness, low surface roughness and moderate corrosion resistance, making it a versatile solution for industrial injection moulds requiring both durability and dimensional precision. Among the analysed systems, the CrN-based BALINIT® CROMA PLUS coating exhibited the highest corrosion resistance, maintaining surface integrity throughout the entire incubation period, while also ensuring stable tribological and optical performance. This confirms its suitability for corrosion-critical applications. Furthermore, a clear correlation between surface roughness and tribological behaviour was identified, with lower Ra values leading to reduced and more stable coefficients of friction, particularly for the 1.2378 HH steel substrate, indicating superior coating–substrate compatibility. The comparison of HV1 and HV0.1 measurements highlights the importance of distinguishing between composite hardness and intrinsic coating hardness. Although HV1 reflects the load-bearing response of the coating–substrate system, HV0.1 provides a more surface-sensitive assessment of coating properties, which is essential for thin PVD coatings where substrate influence is significant.

Overall, the findings confirm that the rational selection of coating–substrate combinations is critical for optimizing tool performance in polymer injection moulding applications. BALINIT® CROMA PLUS is recommended for environments requiring high corrosion resistance, BALINIT® LUMENA for balanced multifunctional performance and BALITHERM® PRIMEFORM for applications dominated by mechanical load. The study provides a comprehensive framework for multi-criteria coating selection, contributing to improved durability, reliability and functional stability of industrial mould components.

## Data Availability

The data presented in this study are available at the Mendeley Data repository at doi:10.17632/vrj72p6pt9.1. Mendeley Data link: https://data.mendeley.com/preview/vrj72p6pt9?a=141fdf48-8b6d-46d5-bfcb- 80854fedcc07.
